# Electrochemical Valence‐Regulated Biomimetic Nanozymes for Breast Cancer Metabolism Inhibition and Potentiated Catalytic Immunotherapy

**DOI:** 10.1002/exp2.70224

**Published:** 2026-09-17

**Authors:** You Pan, Wei Cai, Meng Suo, Xiangqin Meng, Shengxin Ye, Yuping Tan, Xianqing Wei, Mingpu Yang, Pengyuan Qi, Zihang Yu, Kelong Fan, Shipeng Ning

**Affiliations:** ^1^ Department of Breast Surgery The Second Affiliated Hospital of Guangxi Medical University Nanning China; ^2^ Department of Radiology Tongji Hospital, Tongji Medical College, Huazhong University of Science and Technology Wuhan China; ^3^ Research Centers of Nanomedicine Technology & Clinical Medicine The Second Affiliated Hospital of Guangxi Medical University Nanning China; ^4^ CAS Engineering Laboratory For Nanozyme State Key Laboratory of Biomacromolecules Institute of Biophysics Chinese Academy of Sciences Beijing China; ^5^ Cancer Center Union Hospital, Tongji Medical College, Huazhong University of Science and Technology Wuhan China; ^6^ Department of Biomedical Engineering Columbia University New York City New York USA

**Keywords:** biomimetic MnO_x_ nanozymes, catalytic immunotherapy, cGAS‐STING pathway, electrochemical valence regulation, PD‐L1 antagonist

## Abstract

Manganese oxide (MnO_x_) nanozymes have garnered significant attention for their ability to respond to the tumor microenvironment (TME) and stimulator of interferon genes (STING) pathway activation. However, they often suffer from a weak direct tumor‐killing capacity due to low enzymatic activity and from attenuated anti‐tumor immunity resulting from STING activation‐induced excessive programmed death‐ligand 1 (PD‐L1) expression. In this study, we fabricated MnO_x_ nanozymes with precisely controllable valence states via electrochemical valence regulation to modulate their oxidase‐like activity. Notably, M‐MnOx, with a bulk Mn oxidation state of +3.62, a near‐surface Mn oxidation state of approximately +3.23, 19.1% oxygen vacancies, and a reduced Mn−O coordination number of 4.7, exhibited the highest oxidase‐like activity and was therefore selected for cancer therapy. This nanozyme was subsequently wrapped with a T lymphocyte membrane (TCM) to form biomimetic nanozymes (TMO). TMO efficiently targets tumors and induces immunogenic cell death via oxidase catalytic therapy, while also activating the cGAS‐STING pathway and T cells for tumor killing. Furthermore, TMO competitively binds to tumor cell highly expressed PD‐L1 to alleviate T cell immune suppression. Consequently, TMO demonstrates excellent tumor‐killing efficacy and inhibits recurrence in vivo. This research offers a new perspective for the construction of manganese‐based nanomaterials.

## Graphical abstract

MnOx nanozymes face low tumoricidal activity and PD‐L1‐mediated immune attenuation. Electrochemically regulated M‐MnOx with high oxidase activity was TCM‐wrapped into TMO. TMO targets tumors, triggers ICD, activates cGAS‐STING, antagonizes PD‐L1, and suppresses tumor recurrence, providing new insights for Mn‐based nanomaterials.

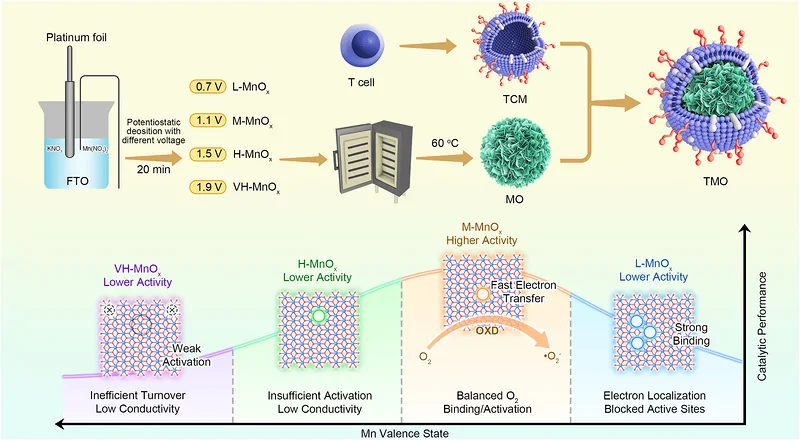

## 1 Introduction

Nanozymes, which integrate the properties of nanomaterials and enzymatic activity, have advantages such as high stability and targeting ability [1, 2, 3, 4, 5, 6]. They can leverage the characteristics of the tumor microenvironment (TME), such as high H_2_O_2_ levels and acidity. Manganese oxide nanozymes, a typical type of functional nanomaterial with enzyme‐mimetic activity, have attracted considerable attention in tumor therapy because of their unique advantages [7, 8]. The core advantage lies in the precise response to and multidimensional regulation of the TME. Specifically, acidic conditions and high concentrations of H_2_O_2_ in the TME can trigger the degradation of MnO_x_ nanozymes, which simultaneously catalyze the decomposition of H_2_O_2_ to produce oxygen and neutralize acidity [9] This not only relieves tumor hypoxia but also attenuates the tumor‐promoting effects induced by acidic microenvironments. Additionally, the manganese ions released after their degradation can induce a Fenton‐like reaction and activate the STING pathway simultaneously, further killing tumor cells and enhancing immunotherapy [10, 11]. However, manganese dioxide nanozymes have a relatively weak ability to directly kill tumor cells and mainly exert auxiliary functions. Therefore, enhancing their direct tumor‐killing capacity to expand their application potential is urgently needed.

To address the above issue, a more meaningful direction is to develop a reliable approach that can precisely tune and control the activity in a controlled way so that the catalytic output can be adjusted to an appropriate therapeutic range. In this context, the key challenge for precise regulation lies in its crystal framework. MnOx (MO) is built from connected [MnO_6_] octahedra, and the lattice is already highly coordinated, which makes it difficult to create and stabilize the active adsorption/activation sites for efficient catalysis [12, 13]. This means that the useful adsorption/activation sites are mainly surface and near‐surface low‐coordination Mn and oxygen vacancies. In practice, however, the Mn valence state (Mn^3^^+^/Mn^4^^+^) and oxygen vacancies are strongly coupled: changing one usually drags the other, and both too many and too few vacancies can hinder electron transport and weaken O_2_ activation [14, 15].

Electrochemical regulation offers a clean, continuous, and quantitative method to tune oxide valence states and defect density (often mainly at the surface/near‐surface) under mild conditions [16, 17]. For example, Lu et al. demonstrated that electrochemical tuning (via controlled delithiation) can continuously reshape the electronic structure of layered transition‐metal oxides and markedly improve oxygen evolution catalysis [18]. Furthermore, Mn–O coordination chemistry is also directly regulated by these processes. Chan et al. reported that Mn^3^^+^ can be introduced into MnO_2_ films through electrochemically induced reactions and persists during operation, accompanied by reduced Mn–O coordination and improved catalytic performance [19]. We expect that electrochemical regulation can offer the following advantages: (i) stepwise control of the Mn^3^^+^/Mn^4^^+^ ratio, (ii) controllable tuning of oxygen vacancies, mainly at the surface and near‐surface where active sites are located, and (iii) a clear link between valence/defects and catalytic activity, rather than trial‒and‐error screening.

Another concern with MnO_x_ is that STING activation can induce PD‐L1 upregulation and impair T‐cell function, thereby limiting the efficacy of Mn^2^^+^/STING‐based immunotherapy [20, 21]. Stimulators of the STING pathway, key regulators of innate immunity, play pivotal roles in antitumor immunity [22]. Its activation is triggered by cyclic dinucleotides (CDNs), such as cGAMP, which bind to the STING protein on immune cells, initiating downstream signaling to induce type I interferons and proinflammatory cytokines [23]. These molecules recruit and activate cytotoxic T cells, natural killer cells, and dendritic cells, resulting in a robust antitumor immune response [24]. Current activation strategies include direct CDN delivery and STING agonist‐based approaches that enhance targeting and stability. Notably, as mentioned, Mn^2^^+^ released from MnO_x_ nanozymes can also activate STING, synergizing with other therapies [10]. Recent studies have demonstrated that activation of the STING pathway can induce aberrantly high expression of PD‐L1 on tumor cell surfaces [21, 25]. This upregulated PD‐L1 binds to programmed cell death protein 1 (PD‐1) on infiltrating cytotoxic T cells, triggering immune checkpoint signaling that impairs T‐cell function. Consequently, this STING‐induced increase in PD‐L1 expression significantly restricts the immunotherapeutic efficacy of MnO_x_ nanozymes, which rely on STING activation to elicit antitumor immune responses.

**SCHEME 1:**
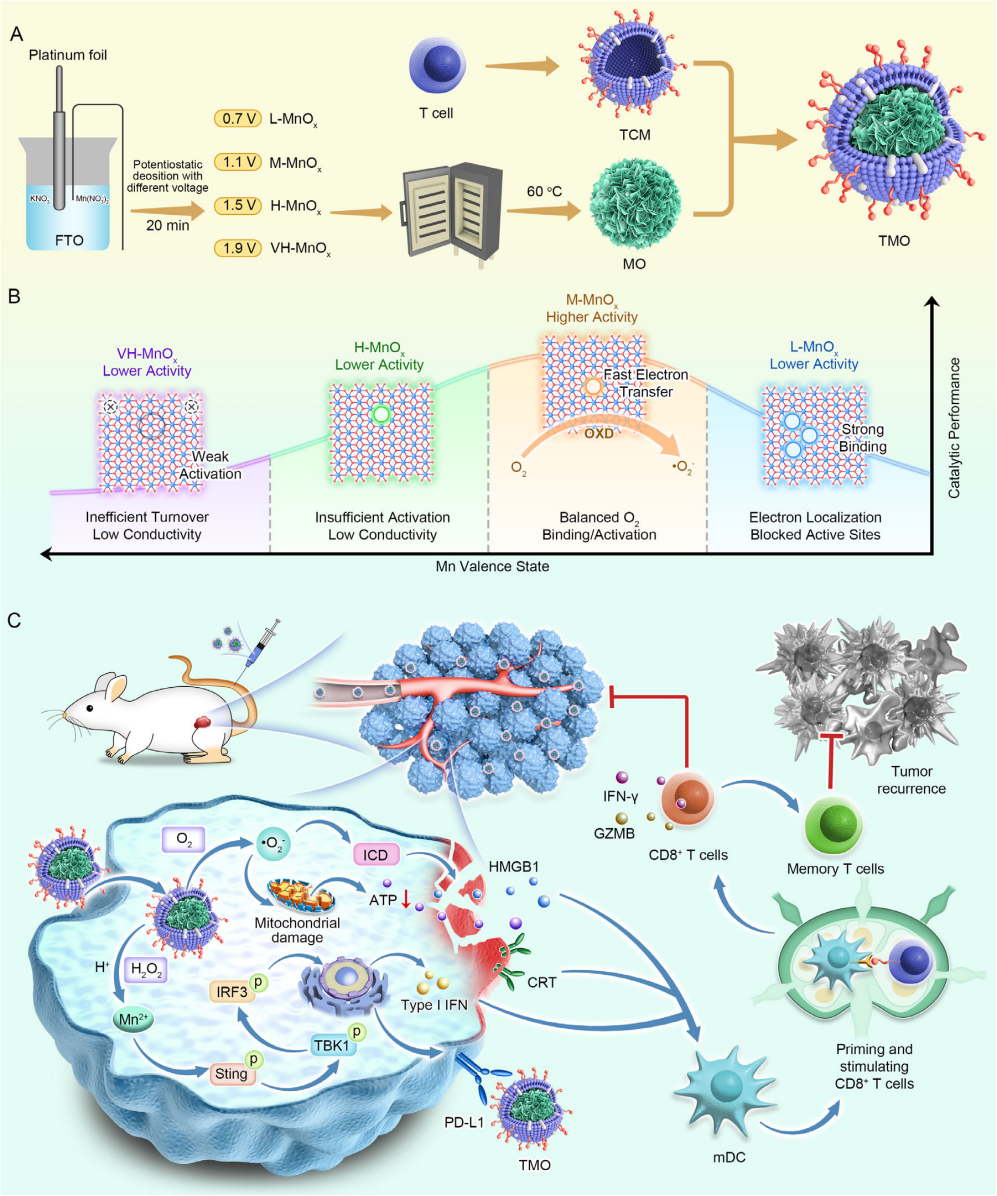
Schematic illustration of electrochemical valence‐regulated biomimetic nanozymes for breast cancer metabolism inhibition and potentiated catalytic immunotherapy.

In this study, we fabricated MO nanozymes with precisely controllable valence states via a systematic electrochemical valence regulation strategy. On this basis, we further explored the intrinsic regulatory factors dominating their enzymatic catalytic performance. By clarifying the synergistic and antagonistic effects between the Mn^3^^+^/Mn^4^^+^ ratio and oxygen vacancies, we revealed that their oxidase (OXD)‐mimetic activity does not increase monotonically with increasing valence state. Instead, it is constrained by the reduced electron delocalization and excessive ordering induced by high valence states. We quantified the relationship between the valence state of M‐MnO_2_ and its activity and analyzed the underlying mechanism. Both experimental and theoretical calculations demonstrated that a sample with a moderate valence state achieves optimal O_2_ adsorption and electron transfer by forming approximately 19.1% oxygen vacancies and tuning the Mn d‐band center to −1.5 eV. We subsequently performed biomimetic modification of the surface of this novel MO nanozyme by wrapping a layer of T lymphocyte membrane (TCM). High‐PD‐1‐expressing TCMs represent a unique type of cell membrane capable of targeting tumor tissues. Unlike other cell membranes, TCMs not only target tumor cells but also competitively block PD‐L1‐mediated T‐cell exhaustion, thereby functioning similarly to anti‐PD‐L1 antibodies. Thus, the overexpression of PD‐L1 mediated by cGAS‐STING pathway activation may be effectively addressed, and T cell membrane‐mediated PD‐L1 antagonism may synergize with manganese dioxide nanozymes to achieve efficient immunotherapy. Our experimental results demonstrated that the TCM‐coated biomimetic manganese dioxide nanozyme (designated TMO) can efficiently target tumor tissues and achieve OXD catalytic therapy. It promotes the occurrence of immunogenic cell death (ICD) in tumor cells, activates the cyclic GMP‐AMP synthase (cGAS)‐STING pathway, stimulates the maturation of dendritic cells (DCs), and ultimately activates T cells to kill tumors. Notably, compared with the platelet membrane‐coated biomimetic manganese dioxide nanozyme (designated PMO), TMO exhibited superior tumor‐killing efficacy and an enhanced ability to inhibit tumor recurrence after treatment. Although PMO can also target tumor tissues for OXD catalytic therapy, the high expression of PD‐L1 induced by STING activation limits the subsequent antitumor capacity of T cells. Therefore, TMO innovatively combines OXD catalytic therapy with PD‐L1 antagonistic therapy, which overcomes the issues that MnO_x_ nanozymes lack efficient and direct tumor‐killing ability and immune side effects caused by the activation of the STING pathway induced by MnO_x_ nanozymes. This study provides new insight into the design of manganese‐based nanomaterials (Scheme 1).

## 2 Results and Discussion

### 2.1 Electrochemical Synthesis and Valence Control of MnO_x_

**FIGURE 1:**
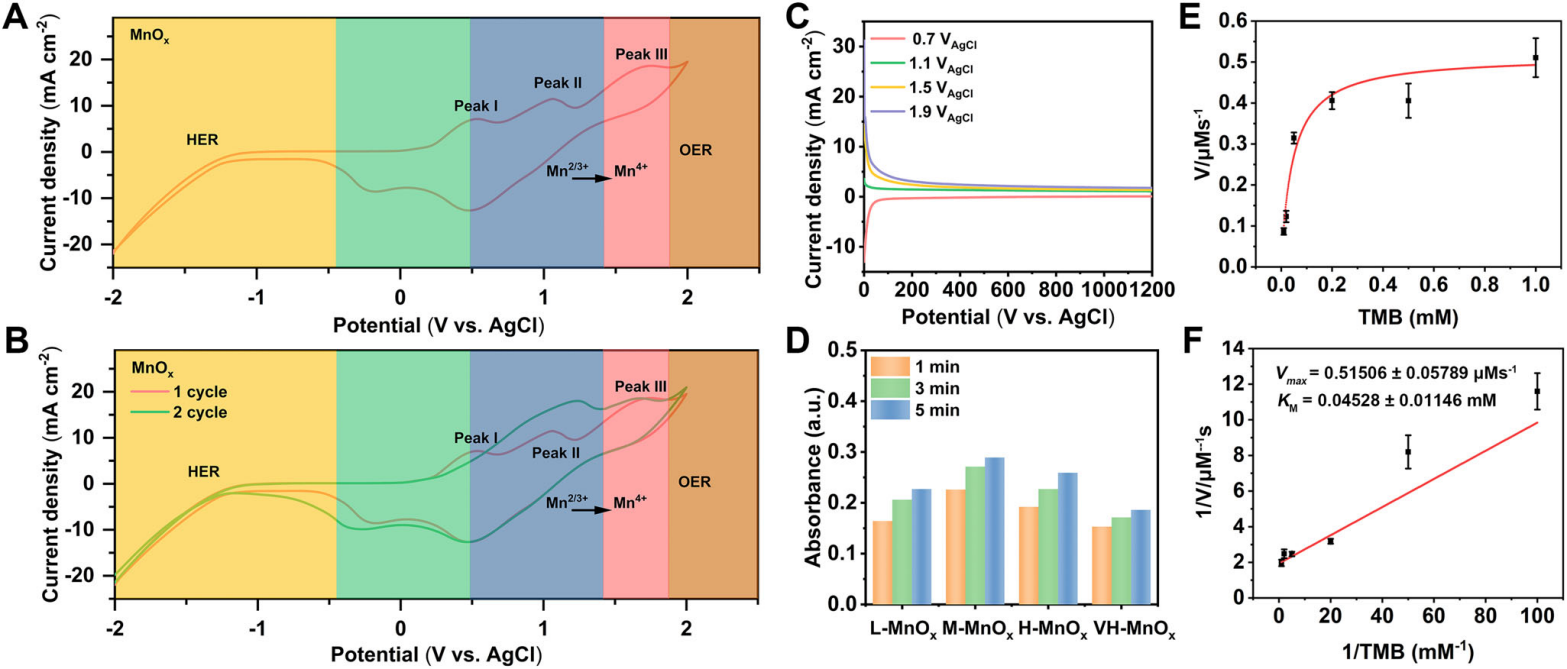
(A) CV curve for MnO_x_ deposition in KNO_3_ and Mn(NO_3_)_2_ solutions. (B) Comparison of the first and second cyclic voltammetry scans for MnO_x_ deposition in KNO_3_ and Mn(NO_3_)_2_ solutions. (C) Chronoamperometric *J–t* curves of MnO_x_ deposition at different applied potentials for 1200 s in KNO_3_ and Mn(NO_3_)_2_ electrolytes. (D) Absorbance at 652 nm of L‐MnO_x_, M‐MnO_x_, H‐MnO_x_, and VH‐MnO_x_ over time. (E) Initial velocity of the reaction with various concentrations of TMB. (F) Double‐reciprocal plots of TMB. The data are shown as the means ± SD (n = 3).

The electrodeposition mechanism of MnO_x_ was systematically investigated via cyclic voltammetry (CV), which revealed a multistep redox process driven by a progressive increase in the applied potential (Figure 1A). As the potential was swept from −0.5 V to 0.5 V, the current density increased linearly because of Mn^2+^ diffusion toward the electrode surface. Upon reaching 0.52 V, the first oxidation peak (Peak I) appeared, indicating the onset of Mn^2+^ → Mn^3+^ oxidation. These unstable Mn^3+^ ions rapidly reacted with water to form Mn(OH)_3_ or MnOOH, which adsorbed onto the Fluorine‐doped Tin Oxide Glass (FTO) surface and established nucleation sites. As the potential increased toward 1.06 V, a more pronounced oxidation peak (Peak II) emerged with approximately twice the current density of Peak I. At this potential, surface−adsorbed Mn^3+^ species were further oxidized to Mn^4+^, while additional Mn^2+^ ions from the solution continued to oxidize and deposit. The 540‐mV potential difference between Peaks I and II reflected the extra energy required for this surface oxidation. When the potential was further increased from 1.4 V to 1.9 V, a broad oxidation feature centered at 1.71 V (Peak III) supported steady film growth, where both the Mn^3+^ and Mn^4+^ oxidation reactions proceeded simultaneously. Beyond 1.9 V, water oxidation (2H_2_O → O_2_ + 4H^+^ + 4e^−^) became dominant, with the deposited MnO_x_ film serving as an oxygen evolution reaction (OER) catalyst. During the reverse potential sweep, two distinct reduction peaks provided insight into the reversibility of manganese redox chemistry. At 0.5 V, Mn^4+^ was reduced back to Mn^3+^, confirming the presence of high‐valence manganese species formed during the forward scan. As the potential decreased further to −0.25 V, Mn^3+^ was reduced to Mn^2+^ and dissolved in the electrolyte. Finally, when the potential was swept to the most negative range (−0.4 V to −2 V), the remaining MnO_x_ film catalyzed the hydrogen evolution reaction (HER). Notably, Peak I disappeared completely in the second cycle, whereas all the other peaks remained unchanged (Figure 1B), indicating that initial Mn^2+^ oxidation occurred on bare FTO, whereas subsequent deposition proceeded on the MnOx‐coated surfaces. This mechanistic insight enabled rational design of valence control strategies. Based on this understanding, we selected four characteristic potentials to precisely control the manganese oxidation state in the MnO_x_ film. At 0.7 V (between Peaks I and II, ≈6 mA cm^−2^), the film was designated low‐valence MnO_x_ (L‐MnO_x_). At 1.1 V (peak II maximum, ≈12 mA cm^−2^), rapid concurrent oxidation and deposition yielded medium‐valence MnO_x_ (M‐MnO_x_). At 1.5 V (Peak III region, ≈15 mA cm^−2^), multiple oxidation processes produced high‐valence MnO_x_ (H‐MnO_x_). At 1.9 V (near the OER onset, ≈18 mA cm^−2^), the highly oxidizing conditions generated very high‐valence MnO_x_ (VH‐MnO_x_).

Chronoamperometric measurements at these four potentials revealed similar deposition kinetics (Figure 1C). At all potentials, the deposition current density exhibited an extremely high initial spike, which was attributed to rapid oxidative nucleation at active sites on the FTO surface and charging of the electrical double layer. Following this transient response, the current gradually decreased and approached a quasi‐steady state after approximately 5 min, reflecting the progressive coverage of the FTO surface by the growing MnO_x_ film. After 20 min of deposition, the current densities at all four potentials converged to similarly low values, indicating that the available active sites on the FTO substrate were largely covered and that the film growth rate substantially decreased. At this stage, the applied potential mainly regulated the manganese oxidation state rather than promoting further growth. We therefore selected 20 min as the optimal deposition time.

To evaluate the influence of valence regulation on catalytic performance, we assessed the OXD‐like activity of MnO_x_ samples with different valence compositions using 3,3',5,5'‐tetramethylbenzidine (TMB) as the chromogenic substrate in a phosphate buffer solution (pH 6.5) [3, 26, 27]. All the samples efficiently catalyzed TMB oxidation, generating a characteristic absorption peak at 652 nm. Furthermore, ABTS was also used as an additional substrate under different pH conditions (pH 5.0, 6.0, and 7.0). The results (Figure S1) were consistent with the TMB assay and showed better activity under weakly acidic conditions, which is favorable for tumor therapy. Strikingly, the catalytic activity exhibited a pronounced volcano‐type dependence on the manganese valence state (Figure 1D). After 1 min of reaction, M‐MnO_x_ had the highest absorbance (0.23), significantly exceeding those of L‐MnO_x_ (0.16), H‐MnO_x_ (0.19), and VH‐MnO_x_ (0.15). This activity trend persisted at 3 min and 5 min, confirming an optimal valence window for maximum catalytic efficiency. The nonmonotonic activity reveals that multiple structural factors collectively govern the catalytic performance, prompting further investigation of their electronic and coordination structures. The obtained Michaelis‒Menten parameters of M‐MnO_x_ (Figure 1E,F) indicate a *K*_M_ value of 0.04528 ± 0.01146 mM and a *V_max_* of 0.51506 ± 0.05789 µM/s for TMB decomposition, which is much better than previously reported oxidase‐like nanozymes [28, 29].

### 2.2 Structural and Electronic Properties of Valence‐Tuned MnO_x_

**FIGURE 2:**
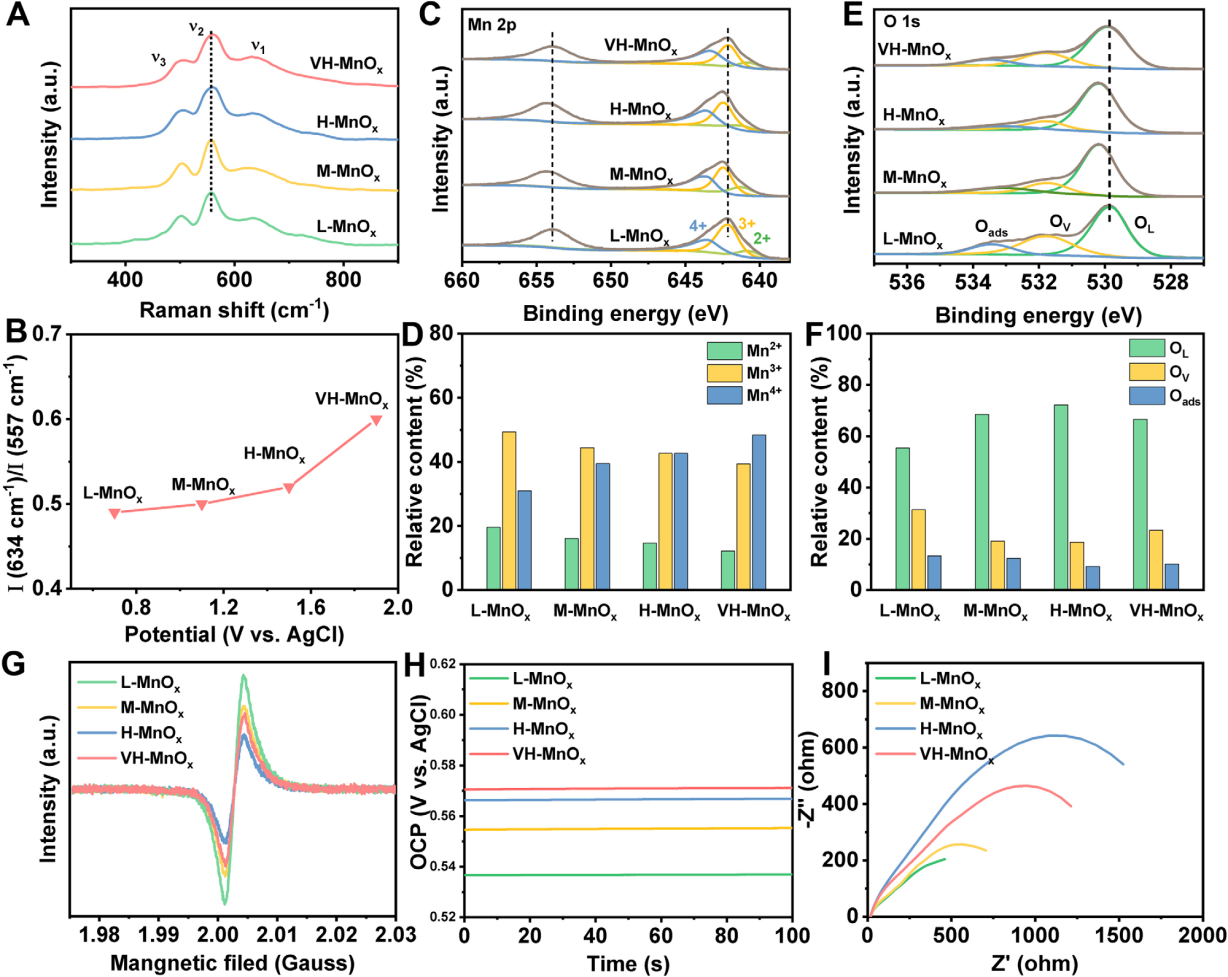
(A) Raman spectra of L‐MnO_x_, M‐MnO_x_, H‐MnO_x_, and VH‐MnO_x_. (B) ν_1_/ν_2_ intensity ratios obtained from Raman peak deconvolution. (C) High‐resolution XPS Mn 2p spectra. (D) Quantitative distribution of Mn^2+^, Mn^3+^, and Mn^4+^ oxidation states from XPS peak fitting. (E) High‐resolution XPS O 1s spectra. (F) Relative contents of O_L_, O_V_, and O_ads_ determined by XPS deconvolution. (G) EPR spectra of L‐MnO_x_, M‐MnO_x_, H‐MnO_x_, and VH‐MnO_x_. (H) OCP measurements of L‐MnO_x_, M‐MnO_x_, H‐MnO_x_, and VH‐MnO_x_, and (I) EIS analysis revealing differences in charge transfer characteristics.

To understand the origin of the volcano‐type activity, we systematically characterized the structural and electronic properties of the valence‐tuned MnO_x_ samples. X‐ray diffraction (XRD) analysis revealed that the diffraction peaks of M‐MnO_x_ were in excellent agreement with the standard PDF card (No. 13–0105), confirming the typical birnessite structure (Figure S2). Birnessite is a layered manganese oxide with edge‐sharing [MnO_6_] octahedra that form 2D framework layers [4, 5]. Interlayer spaces contain water molecules and exchangeable cations (K^+^ and Na^+^) for charge balance, which are held by van der Waals forces. In the octahedral field, Mn 3d orbitals split into lower‐energy t_2g_ (d_xy_, d_yz_, and d_zx_) and higher‐energy e_g_ (d_z_^2^ and d_x_^2^_−y_^2^) orbitals. High‐spin Mn^3+^ (3d^4^ and t_2g_^3^e_g_^1^) exhibits Jahn‐Teller distortion due to the unpaired e_g_ electron, whereas Mn^4+^ (3d^3^ and t_2g_^3^e_g_^0^) maintains better octahedral symmetry. The coexistence of Mn^3+^ and Mn^4+^ creates a mixed‐valence system amenable to electrochemical valence‐state modulation. Raman spectroscopy revealed the impact of Mn valence state changes on local vibrational modes (Figure 2A). All the samples exhibited three characteristic peaks at approximately 634, 557, and 502 cm^−1^, attributed to different vibrational modes of the [MnO_6_] octahedra. The ν_1_ peak at 634 cm^−1^ originates from the symmetric stretching vibration of Mn–O bonds along the c‐axis direction, which primarily involves σ bonding between the Mn 3d_z_^2^ orbitals and O 2p orbitals. The ν_2_ peak at 557 cm^−1^ reflects the asymmetric stretching vibration of in‐plane Mn–O bonds associated with Mn 3d_x_^2^_−y_^2^ orbitals. The ν_3_ peak at 502 cm^−1^ represents the bending vibrational mode of the [MnO_6_] octahedra. As the Mn valence state progressively increased from L‐MnO_x_ to VH‐MnO_x_, the ν_2_ peak position systematically blueshifted from 557 cm^−1^ to 562 cm^−1^, a shift of 5 cm^−1^. This change indicates Mn–O bond length contraction and bond strength enhancement, which can be explained by molecular orbital theory. When Mn^2+/3+^ is further oxidized to Mn^3+/4+^, the Mn 3d orbitals lose an electron, reducing the occupation of Mn–O antibonding orbitals and thereby strengthening the bonding interaction. Simultaneously, the effective nuclear charge of Mn^4+^ exceeds that of Mn^3+^, producing stronger electrostatic attraction toward coordinating oxygen atoms and further contraction of the Mn–O bond. Since the vibrational frequency increases with increasing bond strength, bond contraction leads to a higher vibrational frequency.

To characterize the average Mn oxidation state quantitatively, the peak intensity ratio I(ν_1_)/I(ν_2_) was used as an evaluation metric. The ν_1_ mode is highly sensitive to valence state changes because it involves the d_z_^2^ orbital along the c‐axis, which controls interlayer Mn–O bonding. In contrast, the ν_2_ mode mainly reflects in‐layer vibrations affected by interlayer cations and the layer structure, making it less sensitive to changes in the valence state. Since ν_1_ responds to valence states while ν_2_ serves as a structural reference, their ratio effectively separates valence state effects from structural factors. Figure 2B shows that the I(ν_1_)/I(ν_2_) ratio increased continuously across the sample series, with values of 0.49, 0.50, 0.52, and 0.60 for L‐MnO_x_, M‐MnO_x_, H‐MnO_x_, and VH‐MnO_x_, respectively. This trend is consistent with previous reports showing that the ratio positively correlates with the average Mn oxidation state, confirming that a higher deposition potential effectively promotes Mn oxidation.

To investigate the electronic structure and chemical states, X‐ray photoelectron spectroscopy (XPS) measurements were conducted. The Mn 2p spectra showed that from L‐MnO_x_ to H‐MnO_x_, the Mn 2p3/2 binding energy gradually shifted from 642.1 eV to 642.5 eV (Figure 2C), representing an ∼0.4 eV shift toward higher energy. This shift indicates that Mn oxidation reduces the 3d electron density, weakening electron shielding and thereby increasing the binding energy of the inner 2p electrons. However, VH‐MnO_x_ showed a slight decrease in the Mn 2p3/2 peak at 642.1 eV. This deviation suggests that at high potential, the involvement of lattice oxygen in the OER leads to oxygen vacancy formation, increasing the local electron density around Mn and partially offsetting the oxidation effect [30]. Deconvolution of the Mn 2p3/2 spectra revealed three valence components at 640.7 eV, 642.1 eV, and 643.6 eV, assigned to Mn^2+^, Mn^3+^, and Mn^4+^, respectively. Quantitative analysis (Figure 2D) revealed a systematic oxidation trend, with the Mn^2+^ content progressively decreasing from 19.63% (L‐MnO_x_) to 12.19% (VH‐MnO_x_), whereas the Mn^4+^ content increased from 31.00% to 48.42%. The average oxidation state (AOS) correspondingly increased from +3.11 (L‐MnO_x_) to +3.23 (M‐MnO_x_), +3.28 (H‐MnO_x_), and +3.36 (VH‐MnO_x_). Notably, Mn^3+^ remained stable at ∼45% across all the samples, reflecting the structural requirements of the birnessite framework. The AOS governs the interlayer cation content and structural water, influencing the interlayer spacing and interaction strength. Excessive Mn^4+^ causes interlayer contraction and increased rigidity, whereas appropriate Mn^3+^ introduces lattice distortion via the Jahn–Teller effect, maintaining layer flexibility and ion mobility. This balanced valence distribution stabilizes the layered structure while preserving the structural flexibility essential for catalytic activity.

Changes in the Mn valence state inevitably alter the oxygen coordination environment. Figure 2E presents the O 1 s XPS spectra, which can be deconvoluted into three characteristic peaks at 529.8 eV, 531.2 eV, and 532.5 eV, representing lattice oxygen (O_L_), oxygen vacancies (Ov), and surface‐adsorbed hydroxyl groups (O_ads_), respectively. Lattice oxygen originates from O^2−^ ions directly bonded to Mn within [MnO_6_] octahedra, representing the most stable oxygen species in the system. The lattice oxygen peak gradually shifted from 529.8 eV (L‐MnO_x_) to 530.2 eV (M‐MnO_x_ and H‐MnO_x_) before returning to 529.8 eV in VH‐MnO_x_. Quantitative analysis of the oxygen vacancy concentration revealed a nonmonotonic surface/near‐surface defect trend (Figure 2F). The oxygen‐vacancy content decreased from 31.3% for L‐MnOx to a moderate level for M‐MnOx, reached a minimum of approximately 18.7% for H‐MnOx, and then increased to 23.3% for VH‐MnOx. This trend closely correlates with the evolution of the Mn valence state. In L‐MnO_x_, the high proportions of Mn^2+^ and Mn^3+^ weaken the Mn‐O bonds, as Mn^3+^ (d^4^) electrons occupy antibonding e_g_ orbitals and Mn^2+^ (d^5^) has a low effective nuclear charge, facilitating lattice oxygen desorption and oxygen vacancy formation. As Mn^2+^/Mn^3+^ are progressively oxidized to Mn^4+^ (d^3^) in M‐MnO_x_ and H‐MnO_x_, the e_g_ orbitals become vacant, strengthening Mn−O bonds and suppressing oxygen vacancy formation. Consequently, the oxygen vacancy content in H‐MnO_x_ decreased to its minimum. Notably, the anomalous increase in the oxygen vacancy concentration in VH‐MnO_x_ is consistent with the valence‐state evolution observed in the Mn 2p XPS analysis, further confirming that the lattice oxygen participating in the OER process generated additional oxygen defects. These oxygen vacancies were further confirmed by electron paramagnetic resonance (EPR) spectroscopy. The paramagnetic signal detected at g = 2.002 originates from unpaired electrons associated with oxygen vacancies [2]. As shown in Figure 2G, the signal intensity follows the order of L‐MnO_x_ > M‐MnO_x_ > VH‐MnO_x_ > H‐MnO_x_, which is in excellent agreement with the XPS quantitative results, corroborating the variation trend of the oxygen vacancy concentration from different characterization perspectives. Additionally, the open‐circuit potential (OCP) of the four samples was measured in a phosphate buffer solution (pH 6.5). Figure 2H shows that the OCP values of L‐MnO_x_, M‐MnO_x_, H‐MnO_x_, and VH‐MnO_x_ were 0.54 V, 0.55 V, 0.57 V, and 0.58 V, respectively. The OCP reflects the thermodynamic redox potential of a material [9]. According to the Nernst equation, the OCP is governed by the logarithm of the oxidized‐to‐reduced activity ratio. The increasing proportion of high‐valence Mn ions lowers the Fermi level and enhances the oxidation capability, resulting in an elevated OCP. This OCP evolution trend is highly consistent with the Raman and XPS results. In addition to the thermodynamic redox potential, catalytic performance critically depends on the charge transfer kinetics. We therefore conducted electrochemical impedance spectroscopy (EIS) to assess how valence‐state tuning influences electron transfer rates. Figure 2I shows that the charge transfer resistance initially tends to increase but then decreases. This variation reflects the complex influence of valence states on electron transfer pathways. In low‐valence samples, higher proportions of Mn^3+^ and Mn^2+^ provide more redox‐active sites, resulting in relatively lower impedance. As the Mn^4+^ content increases, the stable d^3^ configuration of Mn^4+^ results in a lower conductivity, leading to increased charge transfer resistance. However, the anomalous decrease in impedance for VH‐MnO_x_ may originate from the participation of lattice oxygen in the OER, which creates additional charge transfer pathways. Moreover, the relatively high oxygen vacancy concentration (15%) increases the carrier concentration, thereby enhancing the electrical conductivity.

### 2.3 Microscopic Structure of M‐MnO_x_

**FIGURE 3:**
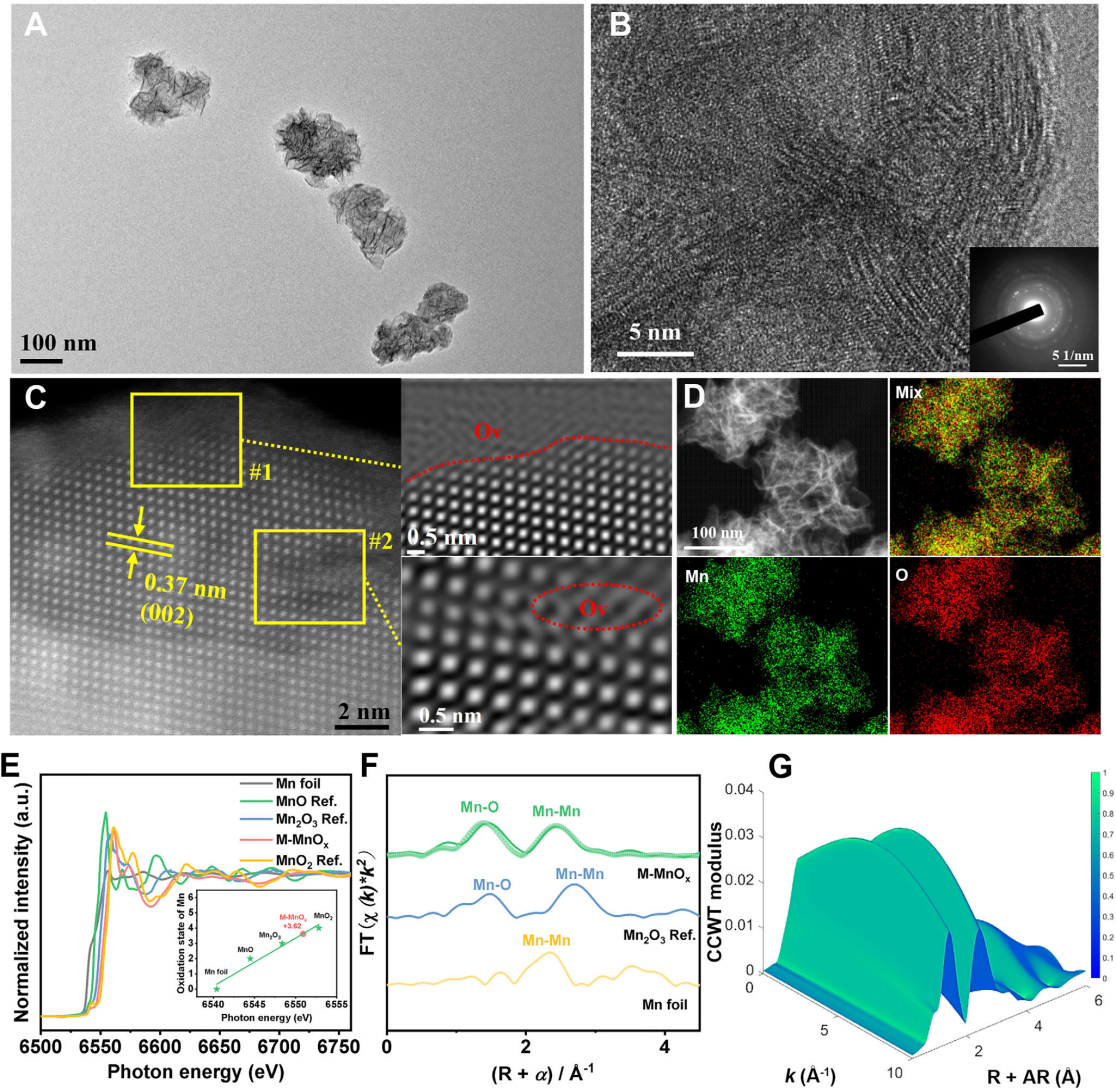
(A) TEM image of M‐MnO_x_. (B) TEM image of M‐MnO_x,_ with the inset showing the corresponding SAED pattern. (C) HAADF‐STEM image of M‐MnO_x,_ with insets showing the associated FFT pattern and IFFT patterns from two selected regions (#1 and #2). (D) Elemental mapping of M‐MnO_x_. (E) Normalized Mn K‐edge XANES spectra of Mn foil, MnO Ref., Mn_2_O_3_ Ref., M‐MnO_x_, and MnO_2_ Ref. The inset shows the average oxidation states of Mn determined from XANES spectra. (F) FFT of Mn K‐edge EXAFS spectra of Mn foil, Mn_2_O_3_ Ref., and M‐MnO_x_. (G) Wavelet‐transformed EXAFS spectra of M‐MnO_x_.

Transmission electron microscopy (TEM) was employed to examine the structure of the most active M‐MnO_x_ sample. As shown in Figure 3A, low‐magnification TEM images reveal that M‐MnO_x_ is composed of ultrathin nanosheets self‐assembled into larger sheet‐like architectures, forming nanospheres constructed from stacked nanosheets. The stacking of these nanosheets generates an open and wrinkled two‐dimensional network, which facilitates the rapid transport of reactants across the interfaces. High‐magnification TEM (Figure 3B) further confirmed that the nanosheets displayed a pronounced layered and corrugated morphology, which was consistent with the typical birnessite‐type layered structure. The ring‐like diffraction patterns in the selected‐area electron diffraction (SAED) pattern indicate relatively low overall crystallinity, implying the presence of abundant structural disorder and oxygen vacancies within the system. Such partial crystallinity may result from the mild electrochemical deposition conditions, which prevent excessive crystallization during high‐temperature annealing and thereby preserve a greater number of catalytically active sites. High‐angle annular dark‐field scanning transmission electron microscopy (HAADF‐STEM) images further revealed the atomic scale structural features of M‐MnO_x_ (Figure 3C). The lattice fringes with an interplanar spacing of ∼0.37 nm correspond to the (002) plane of MnO_2_, confirming that the bulk phase retains a layered manganese oxide framework. Notably, a disordered surface layer approximately 2 nm thick was observed. Inverse fast Fourier transform (IFFT) analysis of regions #1 and #2 revealed locally distorted lattice orientations and amorphous domains, suggesting that oxygen vacancies are highly enriched near the surface. Such surface disorder induced by oxygen defects can reconstruct the Mn–O coordination environment and modulate the electronic structure, thereby promoting surface reaction activity. Elemental mapping (Figure 3D) further revealed a uniform distribution of Mn and O without detectable segregation or enrichment, indicating excellent chemical homogeneity and structural stability.

X‐ray absorption near edge structure (XANES) and extended X‐ray absorption fine structure (EXAFS) techniques were employed to analyze the electronic structures and valence states of the samples. As shown in Figure 3E, the Mn K‐edge absorption of M‐MnOx lies between those of the Mn_2_O_3_ and MnO_2_ standards, corresponding to a bulk‐averaged Mn valence of approximately +3.62 and confirming the presence of mixed‐valence Mn species. This value is higher than the near‐surface AOS of approximately +3.23 obtained from Mn 2p XPS, which can be attributed to the different probing depths of the two techniques. XPS provides surface‐sensitive information, whereas Mn K‐edge XANES reflects the bulk‐averaged electronic structure. Considering the 2 nm oxygen vacancy‐rich disordered layer observed by STEM, the surface Mn valence is expected to be lower than that in the bulk, resulting in a smaller value measured by XPS. The EXAFS spectrum of M‐MnO_x_ exhibited two main peaks at 1.93 Å and 2.82 Å, corresponding to the first Mn–O coordination shell and the intralayer edge‐sharing Mn–Mn neighbors, respectively (Figure 3F). Wavelet transform (WT) analysis further confirmed these two coordination shells and their radial distributions (Figure 3G). In a typical birnessite‐type layered [MnO_6_] framework, the theoretical coordination numbers for Mn–O and intralayer Mn–Mn are 6 and 4, respectively. The EXAFS fitting results for M‐MnO_x_ (Table S1) show that the Mn–Mn coordination number (3.6) is close to the theoretical value, whereas the Mn–O coordination number (4.7) is significantly lower. This reduction in Mn–O coordination agrees well with the presence of an ∼2 nm amorphous surface layer and a high density of oxygen vacancies revealed by XPS and EPR analyses. The oxygen vacancies likely induce partial Mn–O bond cleavage and local coordination reconstruction.

### 2.4 DFT Analysis of the Oxygen Vacancy and Catalytic Performance

**FIGURE 4:**
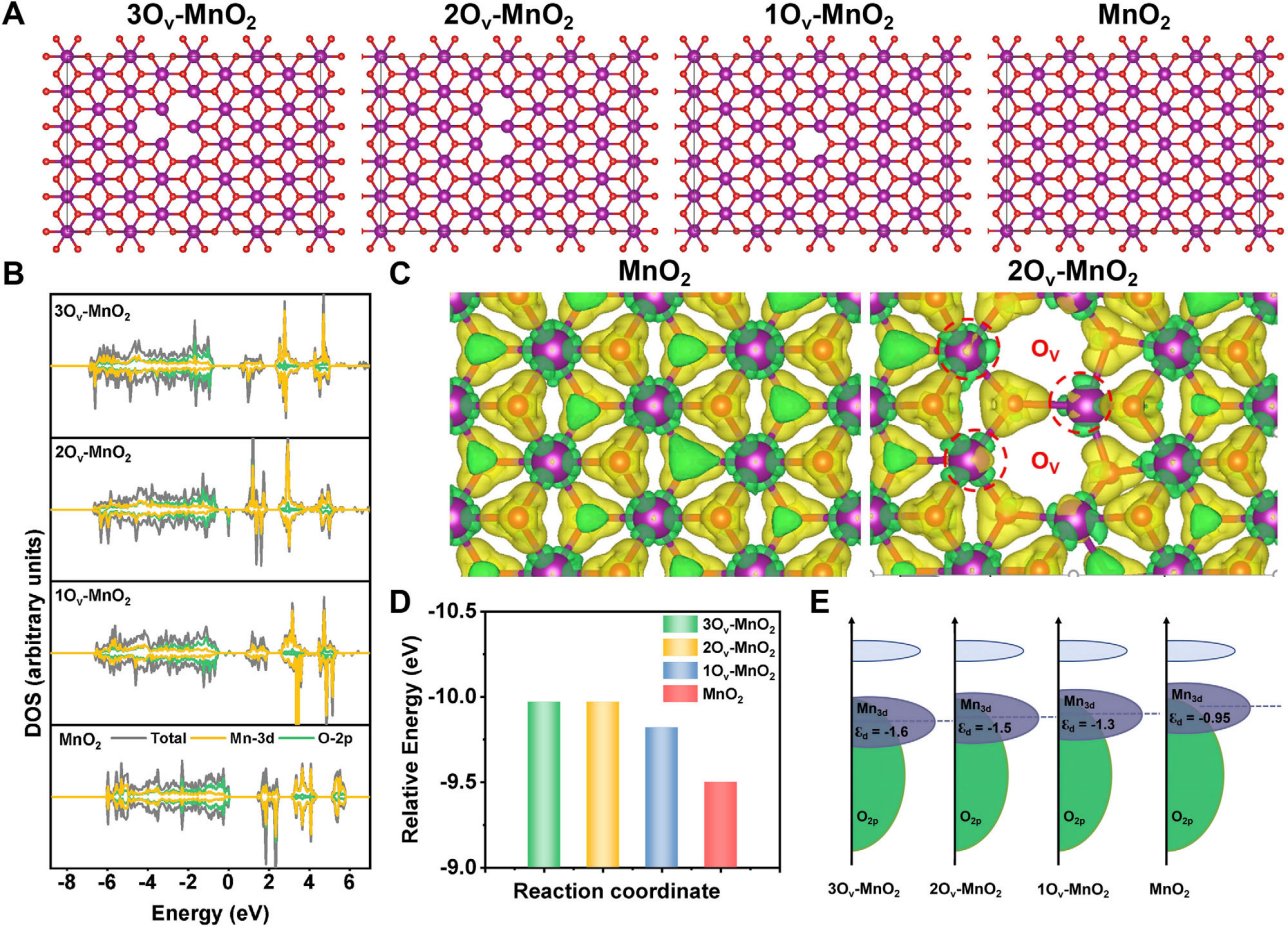
(A) Top views of slab MnO_2_, 1Ov‐MnO_2_, 2Ov‐MnO_2_, and 3Ov‐MnO_2_. (B) DFT calculations of TDOS and PDOS for 3Ov‐MnO_2_, 2Ov‐MnO_2_, 1Ov‐MnO_2_, and MnO_2_. (C) Differential charge density for MnO_2_ and 2Ov‐MnO_2_, where light yellow indicates excess negative charge density and light green represents depletion. (D) Relative energies of O_2_ adsorbed on 3Ov‐MnO_2_, 2Ov‐MnO_2_, 1Ov‐MnO_2_, and MnO_2_. (E) D‐band center of 3Ov‐MnO_2_, 2Ov‐MnO_2_, 1Ov‐MnO_2_, and MnO_2_.

To gain a deeper understanding of how Mn species with different valence states modulate the electronic structure of MnO_x_ and thereby affect their OXD‐like performance, we constructed δ‐MnO_2_ model systems with varying oxygen vacancy (O_V_) concentrations and carried out density functional theory (DFT) calculations (Figure 4A). The model design was guided by the experimentally observed evolution of the average Mn valence state and by previous reports establishing a correlation between oxygen vacancies and Mn oxidation states [10]. Specifically, the formation of oxygen vacancies leads to local electron accumulation, which promotes the reduction of neighboring Mn^4+^ ions to Mn^3+^ or Mn^2+^. Bader charge analysis (Figure **S3** and Table S2) quantitatively confirmed this valence‐state modulation. The density of states (DOS) analysis in Figure 4B reveals that pristine MnO_2_ has typical semiconductor characteristics, with the Fermi level positioned within the band gap. This arises from energy level splitting caused by the t_2g_^3^e_g_° configuration of Mn 3d orbitals under the octahedral crystal field. The introduction of a single oxygen vacancy generated defect states near the Fermi level in 1Ov‐MnO_2_, revealing n‐type doping characteristics and implying enhanced electrical conductivity. Upon increasing to two oxygen vacancies, the DOS of 2Ov‐MnO_2_ at the Fermi level was significantly enhanced, demonstrating that a moderate oxygen vacancy concentration can substantially increase the carrier concentration and electron mobility, thereby optimizing the charge transport kinetics. In contrast, the excessive oxygen vacancy concentration in 3Ov‐MnO_2_ caused local structural distortions and enhanced electron localization, resulting in a decreased DOS at the Fermi level, thus limiting effective electron migration and transfer during reactions. The differential charge density maps in Figure 4C further revealed defect‐induced electron redistribution behavior. Compared with pristine MnO_2_, 2Ov‐MnO_2_ clearly accumulates a negative charge density around the Mn atoms, indicating that oxygen vacancy‐induced electron enrichment enhances the local conductivity. This electron accumulation also provides abundant electrons for active sites, thereby facilitating O_2_ adsorption and activation. Figure 4D shows the relative energies of the O_2_‐bound configurations for pristine MnO_2_, 1Ov‐MnO_2_, 2Ov‐MnO_2_, and 3Ov‐MnO_2_, which are −9.50, −9.82, −9.97, and −9.97 eV, respectively. Note that the energy expression used here does not explicitly subtract the total energy of gas phase O_2_, thus, these values are not standard O_2_ adsorption energies. Instead, they are used only to compare the relative stabilization trend of O_2_‐bound configurations among different oxygen‐vacancy models under the same computational setup. Considering that OXD‐like performance typically proceeds via O_2_ adsorption and activation as the rate‐determining step (RDS), the increased adsorption energy suggests that defect engineering can effectively enhance the catalytic potential of MnO_x_ [11]. The d‐band center analysis in Figure 4E shows that as the oxygen vacancy concentration increased, the Mn 3d band center shifted progressively downward from −0.95 eV (pristine MnO_2_) to −1.3 eV (1Ov‐MnO_2_), further to −1.5 eV (2Ov‐MnO_2_), and −1.6 eV (3Ov‐MnO_2_), indicating that oxygen vacancies modulate the Mn−O bonding character and induce redistribution of d electrons, thereby tuning the O_2_ adsorption propensity. This downshift arises from oxygen vacancy‐induced coordination changes. Oxygen removal weakens Mn−O antibonding interactions, causing the Mn 3d band center to shift away from the Fermi level, with occupied states concentrating toward lower energy regions. According to d‐band center theory, the d‐band center position of transition metals directly determines the strength of bonding‐antibonding orbital interactions with adsorbate species [12]. In 2Ov‐MnO_2_, the Mn d‐band center reaches an appropriate position at approximately −1.5 eV, suggesting that moderate oxygen vacancies modulate the Mn 3d electronic structure. Together with the enhanced DOS near the Fermi level, this feature may provide a favorable electronic environment for interface electron transfer in 2Ov‐MnO_2_, which is consistent with the experimentally observed higher OXD‐like activity.

The OXD‐like activity of MnO_x_ arises from the balance between the O_2_ adsorption strength and electron transfer capability. High‐valence Mn exhibits weak O_2_ adsorption due to the limited number of electrons available for back‐donation, hindering O_2_ activation. In contrast, low‐valence Mn leads to overly strong O_2_ adsorption, difficult product desorption, and pronounced electron localization that inhibits catalytic turnover.

Moderate oxygen vacancies (as in 2Ov‐MnO_2_), corresponding to intermediate Mn valence, optimally position the d‐band center, achieving effective O_2_ π* activation and moderate O−O bond weakening. This will simultaneously balance O_2_ adsorption/activation thermodynamics and interfacial electron transfer kinetics, as evidenced by effective O_2_ π* activation, moderate O–O bond weakening, and an enhanced density of states near the Fermi level. All of these are consistent with the experimentally observed volcano‐type relationship between Mn valence and catalytic performance. This volcano‐type activity reflects a dual electronic requirement, involving a sufficient density of states at the Fermi level to ensure electron transport, along with an appropriate d‐band center to activate O_2_. M–MnO_x_ simultaneously satisfies both conditions, which explains its superior OXD‐like performance.

### 2.5 Preparation and Characterization of T‐cell membrane (TCM)‐coated MO (TMO)

**FIGURE 5:**
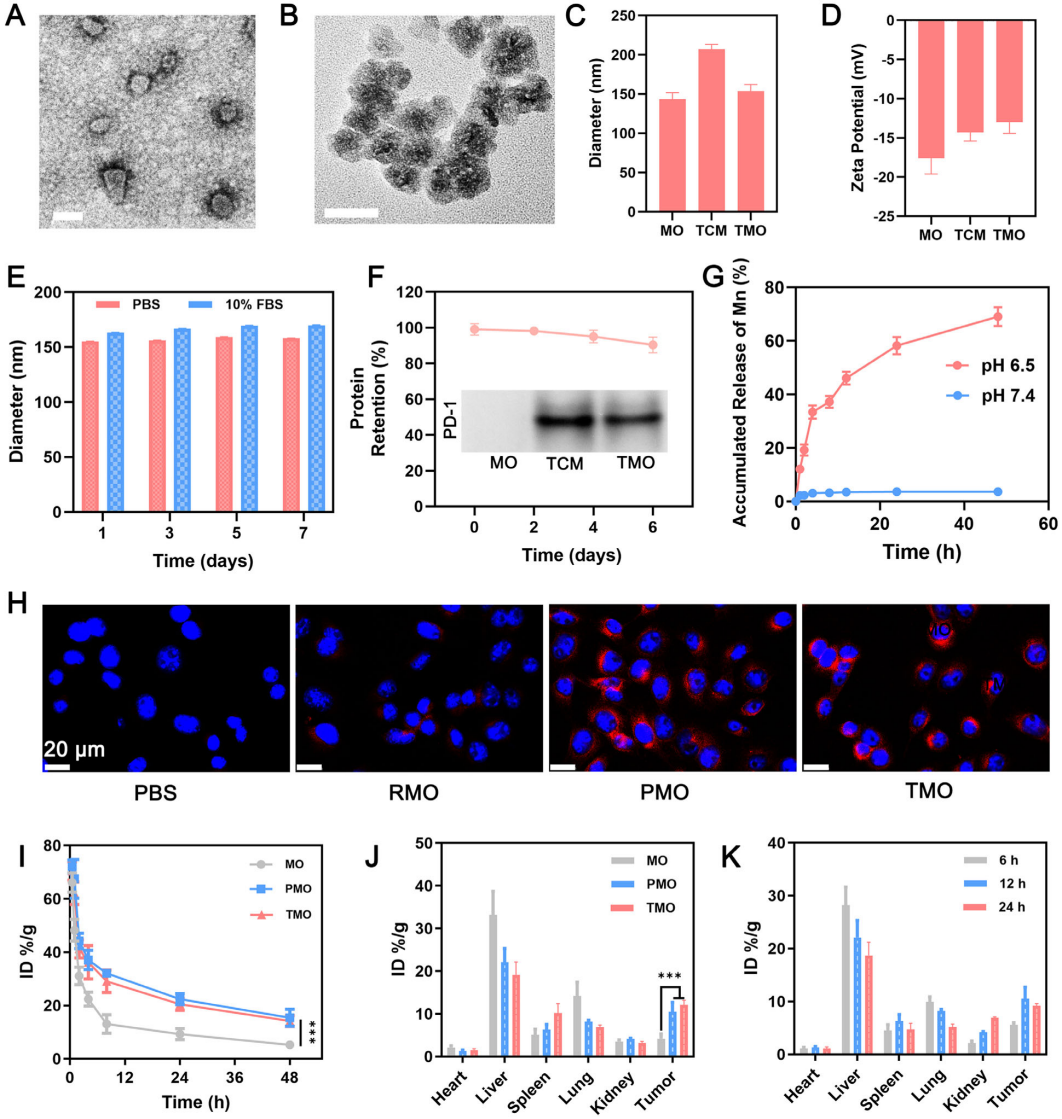
TEM images of the (A) TCM (scale bar: 200 nm) and (B) TMO (scale bar: 200 nm). (C) Diameter and (D) zeta potential of MO, TCM and TMO. (E) Stability of TMO in various solutions at different times. (F) The residual amount of protein in TMO after storage for different periods of time. Insert: Western blot (WB) analysis of PD‐1 proteins in different formulations. (G) Mn release profile of TMO at different pH values. (H) Fluorescence images of cancer cells incubated with DiI‐labeled RMO (red blood cell membrane‐coated MO), PMO (platelet membrane‐coated MO) or TMO for 1 h. Red: Dil; blue: DAPI. (I) Pharmacokinetic curves of MO, PMO and TMO. (J) In vivo biodistribution of the indicated formulations 12 h after injection. (K) In vivo biodistribution of TMO after injection for different durations. The data are shown as the means ± SD (n = 3). Statistical significance was calculated via one‐way ANOVA with Tukey's test: ^***^ *p* < 0.001.

TCM and platelet membranes (PMs) were extracted via previously reported methods, and the MO was encapsulated via physical extrusion to form TMO and PMO. The multidimensional characterization systematically clarified the structural properties, biological behaviors, and in vivo performance of the materials. Transmission electron microscopy (TEM) images revealed that TCM has a regular spherical structure, whereas TMO has a grayish‐white cell membrane structure (Figures 5A and B). The particle size results indicated that the particle size of TMO was greater than that of MO, which can be reasonably explained by the successful coating of TCM on the surface of MO (Figure 5C). The zeta potential results revealed that TCM and TMO have negative potentials with similar values (Figure 5D). We encapsulated MO using PMs to prepare PMO nanoparticles, and the characterization is shown in Figure S4. As a key indicator of dispersion stability, the negative potential of TMOs inherits the surface charge property of TCMs, which is conducive to maintaining their stability in biological systems. Stability and release behavior tests further elaborate the environmental adaptability of the materials (Figure 5E). The particle size shows that TMO has good stability in 10% FBS and PBS. The protein retention rate curve shows that TCM surface proteins can be preserved for a long time, which is beneficial for subsequent biological experiments (Figure 5F). WB results revealed the presence of the PD‐1 protein in TMO. The Mn cumulative release curve revealed a significantly greater release rate at pH 6.5 than at pH 7.4, which reflects the acid‐responsive release characteristics of the materials (Figure 5G). This is a crucial advantage for tumor therapy, as the acidic microenvironment of the tumor can trigger the targeted release of Mn^2^^+^ to enhance the therapeutic effect. We then examined the protein corona adsorption of different materials after blood incubation, as shown in Figure S5. TMO hardly adsorbs protein capsids, thus it does not affect the surface proteins function of the membrane. Cell membrane coating can reduce the protein corona adsorption of nanomaterials [31]. It endows nanomaterials with a biomimetic hydrophilic surface and proper surface charge. This weakens nonspecific electrostatic and hydrophobic interactions with plasma proteins. The cell membrane also acts as a physical barrier to prevent protein adhesion and inhibit protein corona formation. Cell uptake analysis verified the superior cell internalization ability of TMO. In this experiment, for comparison, we introduced an erythrocyte membrane‐coated MO material, namely, RMO. Because there is no protein on the surface of red blood cells that target tumor cells, these cells were used as a control group for the experiments. Confocal microscopy images revealed the cell nuclei (blue) and materials (red), and the stronger red fluorescence of TMO and PMO indicated greater cell uptake efficiency (Figure 5H). To clarify whether the surface functional proteins of TCM remain biologically active after coating treatment, we performed co‐immunoprecipitation (Co‐IP) assays (Figure S6). The results confirmed that the key functional proteins on the TCM surface were stably retained with intact interaction characteristics after coating, demonstrating that the coating modification process would not disrupt the physiological activity of surface functional proteins. To further verify the effective tumor‐targeting capability mediated by TCM surface proteins, we supplemented PD‐L1 antibody blockade combined with immunofluorescence colocalization targeting experiments (Figure S7). The antibody antagonism assay verified that the specific binding between TCM and tumor cells relied on surface functional proteins.

The pharmacokinetic experiments revealed that the TMO and PMO were always higher than the MO over time, indicating that TMO and PMO both have slower clearance rates (Figure 5I). The half‐lives of different nanomedicines are 2.23 h (MO), 7.69 h (TMO), and 10.48 h (PMO), respectively. The organ distribution results revealed that TMO and PMO had significantly greater accumulation in tumors than MO had, with the highest accumulation in the liver (Figure 5J), which may be related to the phagocytosis of the spherical MO by the reticuloendothelial system. The dynamic organ distribution at different time points revealed that maximal tumor accumulation was observed 12 h after injection (Figure 5K). Collectively, these characterization results comprehensively demonstrate that TMO has optimized structural stability, acid‐responsive release properties, efficient cell uptake, and excellent in vivo tumor targeting, which not only verifies the rationality of the TCM coating strategy but also provides a solid foundation for its subsequent antitumor efficacy verification and evaluation of its potential for clinical transformation.

### 2.6 In Vitro Antitumor Ability of TMOs

**FIGURE 6:**
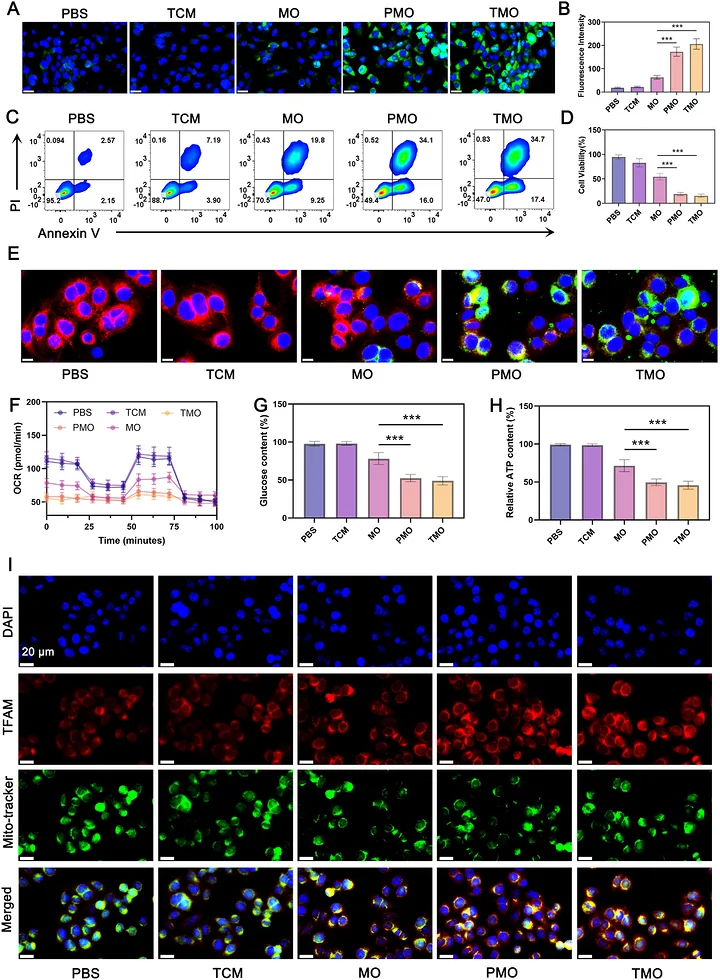
(A) Confocal laser scanning microscopy (CLSM) images and (B) quantitative green fluorescence intensity of 4T1 cancer cells treated with different formulations. The data are shown as the means ± SD (n = 3). DAPI: blue; DHE: green. Scale bar: 20 µm. (C) Flow cytometry analysis of apoptosis after treatment with different formulations. (D) The viability of 4T1 cells after various treatments with different formulations. The data are shown as the means ± SD (n = 3). (E) Mitochondrial membrane potential of 4T1 cells after 24 h of incubation with different agents. DAPI: blue; JC‐1 aggregation: red; JC‐1 monomer: green. Scale bar: 10 µm. (F) Oxygen consumption rate (OCR) of 4T1 cells after treatment with different formulations. The data are shown as the means ± SD (n = 3). (G) Intracellular glucose content after 24 h of incubation with different agents. The data are shown as the means ± SD (n = 3). (H) Intracellular ATP levels after treatment with different formulations. (I) Confocal immunofluorescence staining of TFAM expression and mitochondrial localization in 4T1 cells following different treatments. Nuclei were counterstained with DAPI (blue), TFAM protein was detected by red fluorescence, and mitochondria were labeled with Mito‐tracker (green). The data are shown as the means ± SD (n = 3). Statistical significance was calculated via one‐way ANOVA with Tukey's test: ^*^ *p* < 0.05, ^**^ *p* < 0.01, ^***^ *p* < 0.001.

To elucidate the antitumor mechanism of the developed materials, a comprehensive set of biological effect evaluations was conducted, focusing on reactive oxygen species (ROS) generation, apoptosis, immune signaling activation, and immunomodulatory responses across different treatment groups (PBS, TCM, MO, PMO, and TMO). These assessments are rooted in the core paradigm of nanozyme‐based cancer therapy, where catalytically induced oxidative stress and subsequent immunogenic regulation synergistically mediate tumor suppression. ROS generation, a primary driver of oxidative stress‐induced tumor cell damage, was evaluated via DHE fluorescence probing. DHE is a well‐established ROS‐sensitive fluorogenic probe that undergoes oxidation to emit fluorescence, with the signal intensity directly correlated with the intracellular superoxide anion (·O_2_^−^) level. Figures 6A and B clearly show that both the PMO and TMO treatment groups exhibited robust green fluorescence, significantly outperforming the PBS control and TCM groups. The elevated ROS production can be attributed to the oxidase‐like activity of the MnO_2_ nanozymes in MO, PMO, and TMO, which is consistent with our earlier finding that moderately valent MnO_x_ has optimal oxidase activity. The biological consequences of ROS overproduction were investigated via Annexin V/PI double‐staining flow cytometry (Figure 6C), the gold standard for distinguishing early apoptotic, late apoptotic, and necrotic cells. The results revealed that the late apoptosis rate of the PMO and TMO groups was significantly greater than that of the PBS control group. In contrast, the MO group showed only modest apoptosis induction, likely due to its poor tumor targeting ability and stability, highlighting the critical role of the membrane coating in enhancing therapeutic delivery and efficacy. The results of the CCK‐8 assay also revealed that TMO and PMO strongly inhibited tumor growth (Figure 6D). To further verify the mechanisms by which PMO and TMO inhibit tumor growth, we systematically evaluated the changes in mitochondrial function after different treatments. JC‐1 dye, a well‐established probe for monitoring the mitochondrial membrane potential (MMP), exhibits dual fluorescence depending on the MMP status. In healthy mitochondria with a high MMP, JC‐1 forms aggregates that emit intense red fluorescence. In contrast, mitochondrial depolarization leads to the dissociation of JC‐1 aggregates into monomers, resulting in a shift to green fluorescence—a hallmark of MMP loss. As shown in Figure 6E, cells treated with PMO or TMO displayed markedly reduced red fluorescence and increased green fluorescence, leading to a predominant green signal in the merged images. In contrast, the control groups presented strong red fluorescence, indicating a preserved MMP. These results demonstrate that PMO and TMO disrupt mitochondrial membrane potential, thereby inducing mitochondrial dysfunction. In addition, we investigated cellular metabolism after TMO and PMO treatment. The mitochondrial oxygen consumption rate (OCR) was measured with a Seahorse‐XF24, as shown in Figure 6F. Compared with the control cells, the 4T1 cells treated with MO presented lower basal and maximal respiration rates, indicating that MO treatment effectively inhibited the mitochondrial respiratory system. The respiratory reserve capacity stimulated by PMO and TMO also significantly decreased, suggesting that both PMO and TMO could effectively induce mitochondrial dysfunction. Furthermore, to assess the impact of PMO and TMO on the intracellular metabolic network, we quantitatively analyzed the glucose content in tumor cells. As shown in Figure 6G, the addition of MO significantly reduced the glucose content, whereas the addition of PMO and TMO further decreased the glucose content in 4T1 cells by nearly twofold, indicating the disruption of cellular metabolic communication. Additionally, the intracellular ATP level after treatment with different formulations was examined (Figure 6H). As expected, ATP levels in tumor cells were significantly reduced after PMO and TMO treatment, whereas the PBS group presented minimal changes in ATP levels. Immunofluorescence analysis of mitochondrial TFAM expression and localization in cancer cells treated with different formulations (Figure 6I).

**FIGURE 7:**
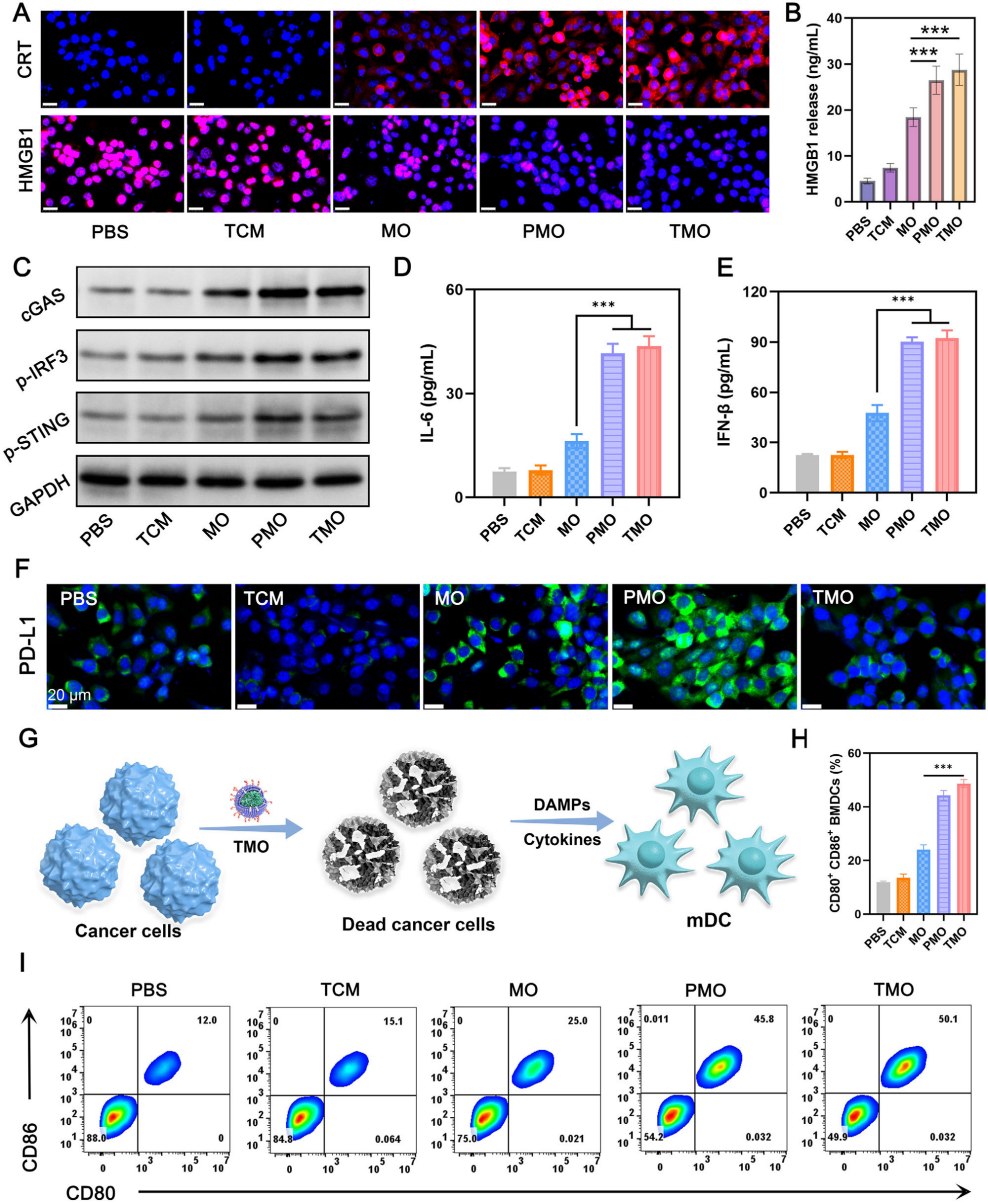
(A) Fluorescence images of CRT and HMGB1 expressions in 4T1 cells after different treatments. scale bars: 20 µm. (B) Analysis of HMGB1 released from 4T1 cells after different treatments. The data are shown as the means ± SD (n = 3). (C) Detection of marker proteins involved in the cGAS‒STING pathway via western blot analysis. (D) ELISA results of IL‐6 and (E) IFN‐β in the medium of 4T1 cells subjected to different treatments. The data are shown as the means ± SD (n = 3). (F) Fluorescence images of PD‐L1 expressions in 4T1 cells after different treatments. (G) Schematic illustration of the mechanism by which TMO induces ICD in cancer cells to promote dendritic cell maturation. (H) quantitative analysis and (I) Flow cytometry analysis of mature BMDCs after different treatments. The data are shown as the means ± SD (n = 3). Statistical significance was calculated via one‐way ANOVA with Tukey's test: ^***^ *p* < 0.001.

To characterize the immunomodulatory profile further, key ICD markers were evaluated (Figures 7A,B and S8). ICD is defined by the release of damage‐associated molecular patterns (DAMPs), including calreticulin (CRT), ATP, and high‐mobility group box 1 (HMGB1) [32, 33, 34, 35]. The results revealed that the PMO and TMO groups presented strong fluorescence signals for both CRT, and the extracellular HMGB1 and ATP levels confirmed that nanozyme treatment induced robust ICD. The antioxidant NAC can inhibit the expression of CRT mediated by TMO, indicating that reactive oxygen species play a major role in this process (Figure S9). Given the importance of ICD and innate immune activation in long‐term antitumor immunity, the activation status of the cGAS‐STING pathway, a key innate immune signaling cascade that senses cytosolic DNA and triggers type I interferon responses, was assessed via Western blotting (Figure 7C). The cGAS‐STING pathway is activated by DNA damage and leads to the phosphorylation of STING (p‐STING) and downstream IRF3 (p‐IRF3), ultimately promoting interferon production. Our results revealed that, compared with the other groups, the PMO and TMO groups presented significantly upregulated expressions of cGAS, p‐STING, and p‐IRF3, with negligible changes in the internal reference protein GAPDH. A potent and selective covalent inhibitor of STING (C178) can inhibit the activation of the cGAS‐STING pathway by TMO (Figure S10). These findings confirm that MnO_x_‐based nanozymes not only induce direct tumor cell apoptosis but also activate the innate immune system by triggering the cGAS‐STING pathway. The upregulation of IFN‐β and IL‐6, chemokines downstream of STING activation, was shown in Figure 7D,E. However, Figure 7F reveals a double‐edged sword: PMO and MO treatment also upregulate the expression of PD‐L1, an immune checkpoint molecule that binds to PD‐1 on T cells to suppress antitumor immune responses [36]. This observation aligns with previous studies showing that STING activation can paradoxically upregulate PD‐L1, highlighting the critical limitations of the PMO and MO. However, owing to the introduction of the T‐cell membrane, the TMO group had very low PD‐L1 expression (Figure S11), which overcame the side effects of this therapy. The impact on innate immunity was assessed by analyzing bone marrow‐derived dendritic cell (BMDC) maturation markers (Figure 7G–I). Flow cytometry scatter plots and quantitative analysis revealed that both the PMO and TMO groups significantly upregulated CD80 and CD86 expression on BMDCs.

In summary, these biological effects reveal a synergistic antitumor mechanism of TMO: (1) TCM‐mediated tumor targeting enhances cellular uptake; (2) oxidase‐like activity induces ROS overproduction, leading to direct tumor cell apoptosis; (3) Mn^2+^‐induced DNA damage activates the cGAS‐STING pathway, promoting innate immune activation; and (4) the TCM coating antagonizes cGAS‐STING pathway activation‐induced PD‐L1 upregulation, preserving T‐cell activation.

### 2.7 In Vivo Antitumor Ability of TMOs

**FIGURE 8:**
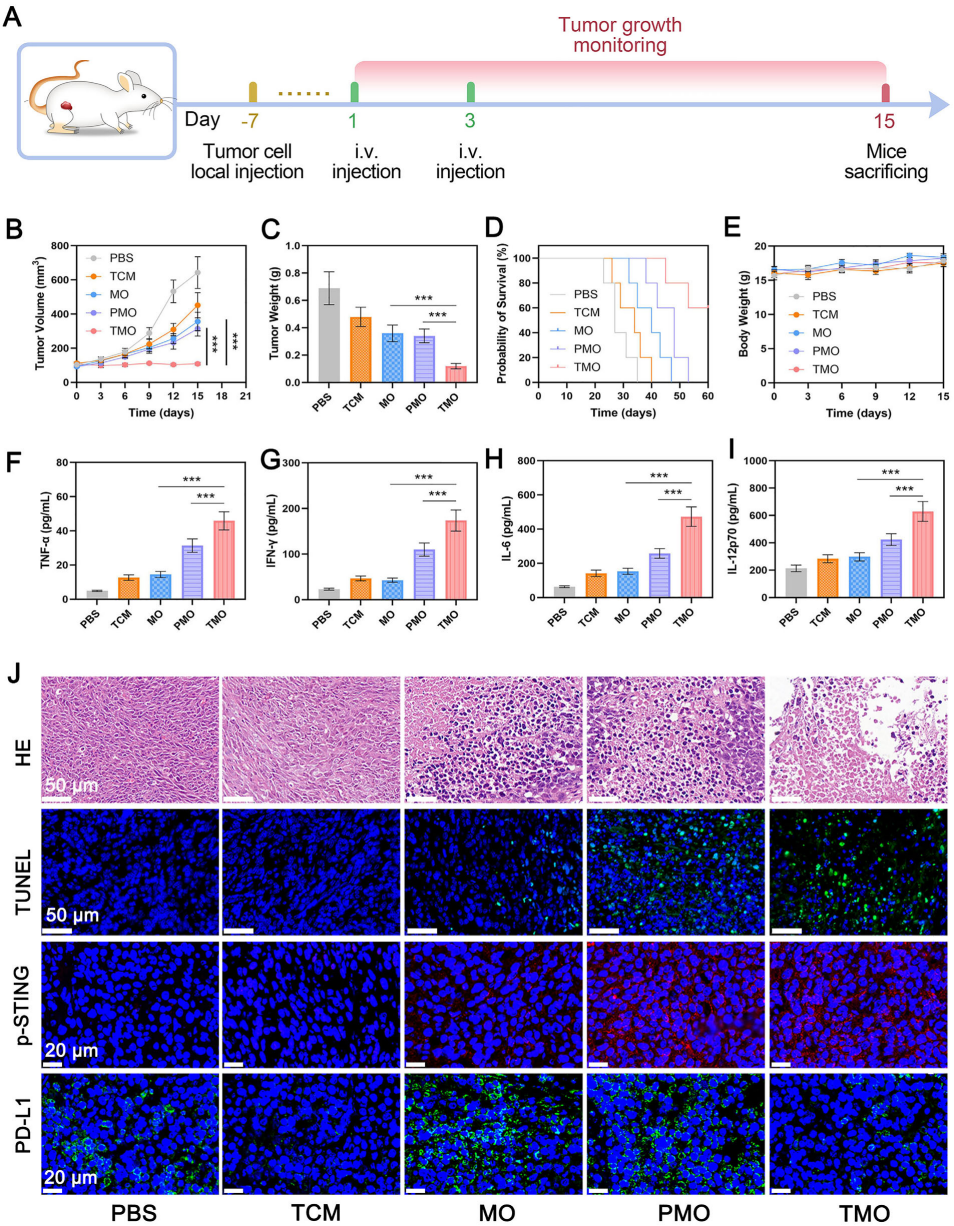
(A) Schematic illustration of the studies of 4T1 tumor therapy. (B) Changes in tumor volume and (C) tumor weight after various treatments. The data are shown as the means ± SD (n = 5). (D) Survival curves after treatment. (E) Changes in body weight during the treatment period. The data are shown as the means ± SD (n = 5). (F) ELISA measurements of the intratumoral secretion of TNF‐𝛼, (G) IFN‐𝛾, (H) IL‐6, and (I) IL‐12p70. The data are shown as the means ± SD (n = 5). (J) HE, TUNEL, p‐STING and PD‐L1 staining of tumor sections after the indicated treatments. Statistical significance was calculated via log‐rank test, one‐way ANOVA, or two‐way ANOVA: ^***^ *p* < 0.001.

**FIGURE 9:**
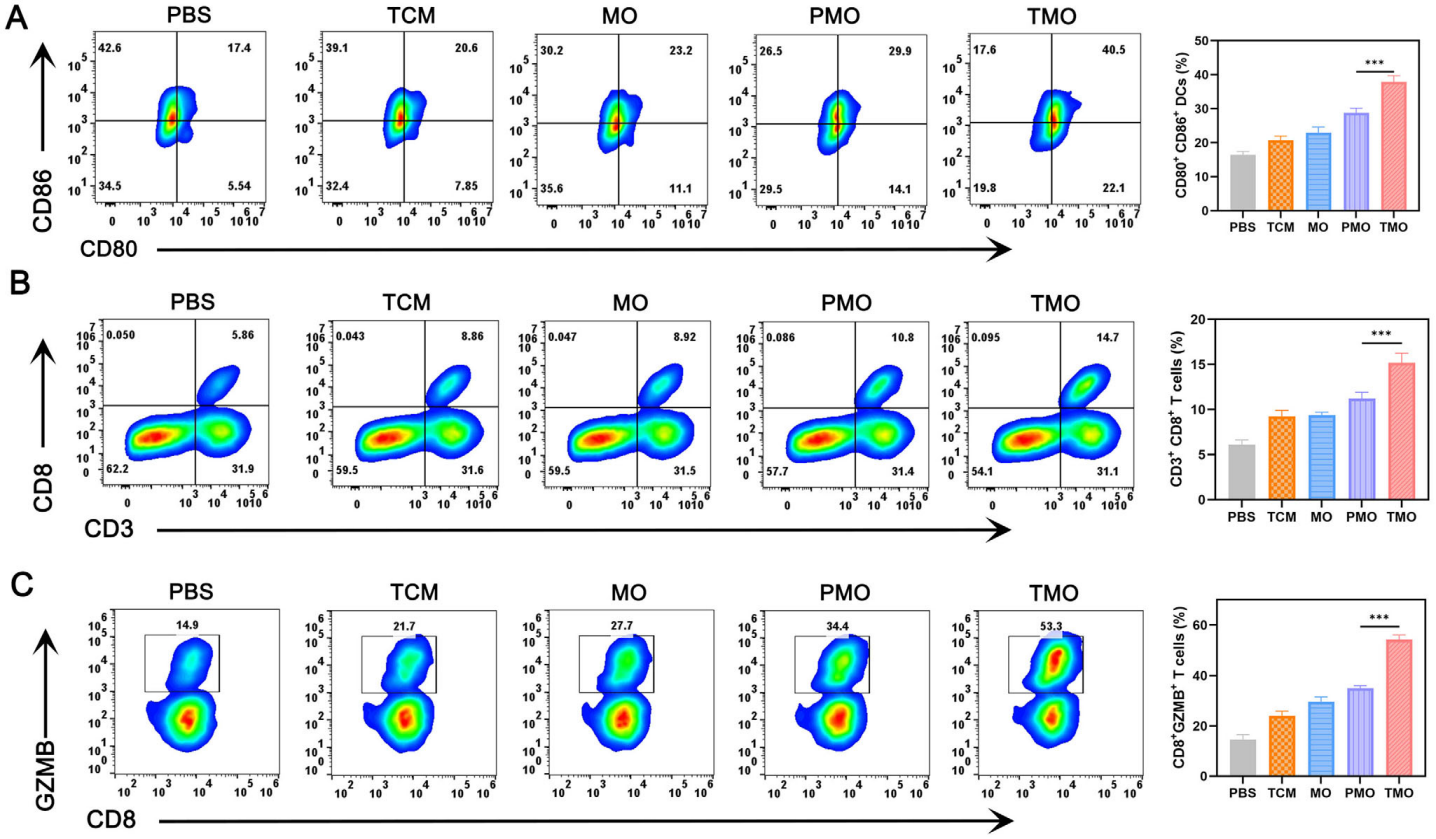
(A) Flow cytometry and quantitative analysis showing the effects of treatments on DC maturation in the lymph nodes. The data are shown as the means ± SD (n = 5). (B) Flow cytometry and quantitative analysis showing the infiltration of CD3^+^ CD8^+^ T lymphocytes in 4T1 tumor tissues. The data are shown as the means ± SD (n = 5). (C) Flow cytometry and quantitative analysis showing GZMB^+^ cells among CD8^+^ T cells in tumor tissues. The data are shown as the means ± SD (n = 5). Statistical significance was calculated via one‐way ANOVA with Tukey's test: ^***^ *p* < 0.001.

To comprehensively validate the antitumor efficacy of the developed materials and elucidate their regulatory effects on tumor progression and the TME, a systematic in vivo study was conducted using a mouse tumor model (Figure 8A). Firstly, we investigated the effects of different TMO injection doses on tumor treatment and side effects. As shown in Figure S12,13, an injection dose of 20 mg/kg was safer and resulted in good therapeutic outcomes. In the group treatment, tumor growth kinetics (Figure 8B) and quantitative tumor weight analysis (Figure 8C) collectively confirmed the superior tumor‐suppressive efficacy of TMO. The PBS control group exhibited unimpeded tumor growth throughout the experiment, with a steady increase in tumor volume. In contrast, all the treatment groups (TCM, MO, PMO, and TMO) presented significant tumor growth inhibition, with TMO showing the most prominent effect. The modest efficacy of MO alone further emphasizes the critical role of membrane coating in enhancing therapeutic delivery, as poor targeting and stability limit its in vivo performance. Notably, TCM alone results in weak but measurable tumor inhibition, possibly attributed to the inherent immunomodulatory properties of T lymphocyte membrane components, such as surface antigens that trigger mild immune responses. Survival analysis (Figure 8D) and body weight monitoring (Figure 8E) were used to evaluate the safety and survival benefits of the treatments. After 28 days of treatment, there were no obvious organ damage or changes in biochemical indicators, indicating their excellent biological safety (Figure S14,15). The BMDC cells and T cells of the mice maintained normal functions, and the manganese content in the body decreased rapidly, which also indicates its good biological safety (Figure S16,17). Survival analysis further confirmed the clinical relevance of these findings: the PBS group had the lowest survival rate, with all the mice succumbing to tumors by the end of the observation period. In comparison, TMO achieves the highest survival rate, with a significantly prolonged median survival time compared with those of PMO and MO. This survival benefit is attributed not only to direct tumor cell killing but also to the long‐term immunomodulatory effects of TMO. Cytokine analysis (Figure 8F–I) further clarified the effects of TME remodeling. TMO treatment significantly increases IL‐6, TNF‐α, IFN‐γ, and IL‐12p70 levels, shifting the TIME from an immunosuppressive “cold tumor” state to an immunostimulatory “hot tumor” state. Hematoxylin and eosin (HE) staining revealed that the PBS group presented dense, disorganized tumor cell aggregates with intact cell morphology, whereas the TMO‐treated tumor tissues presented a sparse cell distribution, fragmented nuclei, and extensive necrosis, which was consistent with the tumor volume reduction observed earlier (Figure 8J). TUNEL staining confirmed massive apoptosis in TMO‐treated tumors, with significantly more apoptotic cells than in the PBS group. p‐STING staining revealed strong positive signals in the TMO and PMO groups, confirming the in vivo activation of the cGAS‒STING pathway. This activation is critical for initiating innate immunity and recruiting T cells. Notably, PD‐L1 staining revealed that TMO treatment reduced PD‐L1 expression compared with that in the PMO and PBS groups, confirming the antagonistic effect of the TCM coating on PD‐L1 expression. The remodeling of the immune microenvironment is the core mechanism for achieving long‐term antitumor effects. Notably, we performed tumor tissue hypoxia and mitochondrial membrane permeability assays and found that nanozyme treatment not only failed to promote tumor hypoxia, but also induced mitochondrial damage in tumor cells, ultimately alleviating tumor hypoxia (Figure S18). This indicates that our material can not only treat tumors but also overcome the potential side effects of OXD nanozyme. We then analyzed the regulatory effects of each treatment regimen on immune function through flow cytometric immunophenotyping (Figure 9A–C) and quantitative detection of immune cell populations. The flow cytometry results revealed extremely low activation levels of immune cell subsets in the PBS control group. In contrast, TMO treatments significantly increased the amount of mature DCs, CD8^+^ T cells, and GZMB^+^CD8^+^ T cells. These findings suggest that TMOs can not only promote the activation and proliferation of CD8^+^ T cells but also enhance their killing function.

**FIGURE 10:**
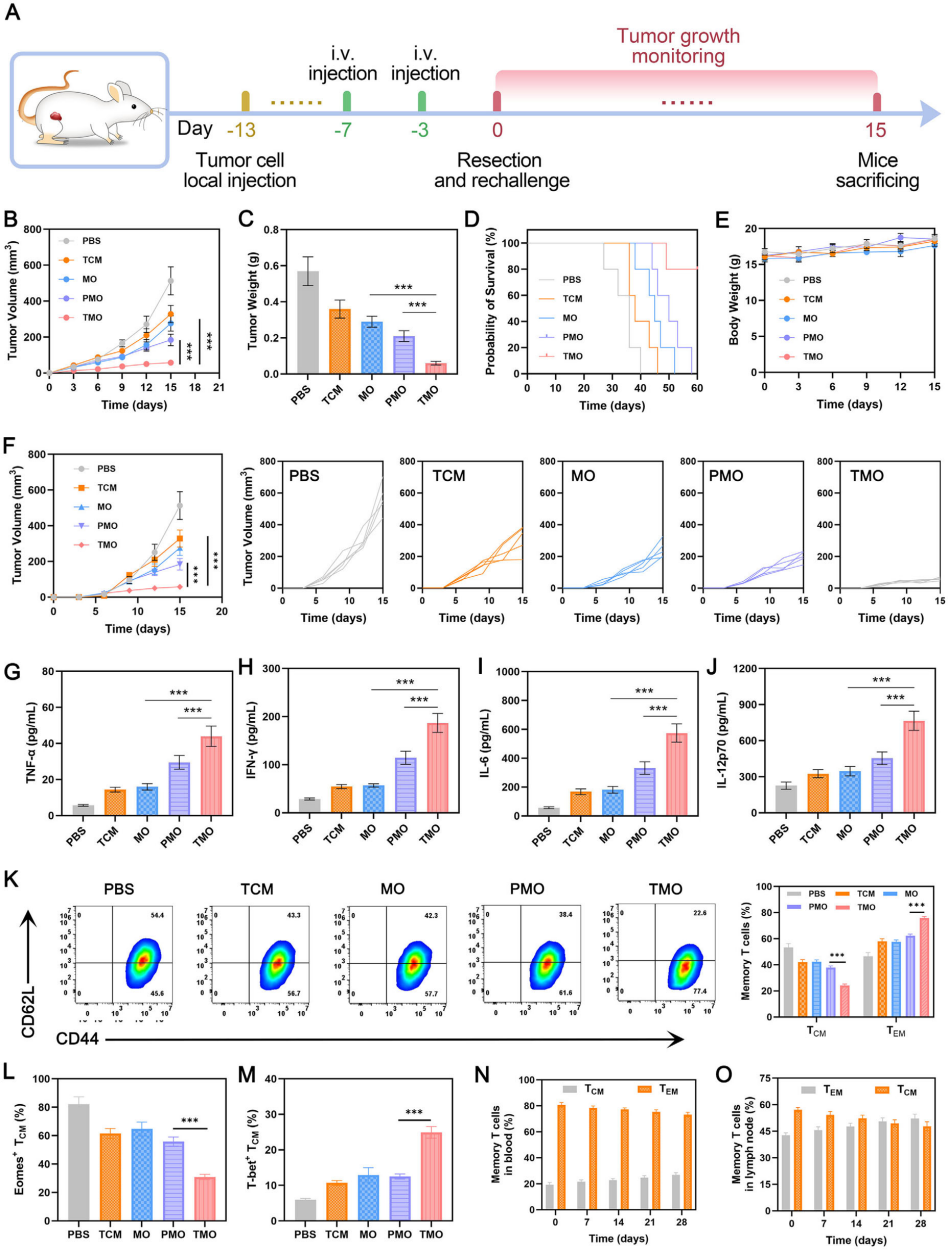
(A) Schematic illustration of recurrent and rechallenged 4T1 tumor therapy. (B) Changes in recurrent tumor volume and (C) tumor weight after various treatments. The data are shown as the means ± SD (n = 5). (D) Survival curves after treatment. (E) Changes in body weight during the treatment period. (F) Changes in rechallenged tumor volume after various treatments. The data are shown as the means ± SD (n = 5). (G) ELISA measurements of TNF‐𝛼, (H) IFN‐𝛾, (I) IL‐6, and (J) IL‐12p70 in the serum. The data are shown as the means ± SD (n = 5). (K) Flow cytometry and quantitative analysis showing memory T cells (T_EM_ and T_CM_) in blood. The data are shown as the means ± SD (n = 5). (L) Frequencies of Eomes+ and (M) T‐bet+ cells in T_CM_ (n = 5). (N) The proportion of memory T cells in the blood and (O) lymph nodes within 28 days after tumor resection. The data are shown as the means ± SD (n = 5). Statistical significance was calculated via log‐rank test, one‐way ANOVA, or two‐way ANOVA: ^***^ *p* < 0.001.

To assess the antitumor recurrence activity of TMO, we employed a recurrent tumor model (Figure 10A). Dynamic monitoring of tumor growth (Figure 10B) and statistical analysis of tumor weight (Figure 10C) confirmed distinct differences in tumor inhibitory efficacy between various treatment regimens. Specifically, after surgical intervention, tumors in the PBS control group exhibited continuous and rapid recurrence and proliferation, with volumes increasing exponentially over time; in sharp contrast, all treatment groups (TCM, MO, PMO, and TMO) showed significant tumor growth inhibition, among which the TMO group achieved the most prominent effect, while the MO group displayed a certain degree of tumor inhibitory activity, and its efficacy was far inferior to that of the PMO and TMO groups. Survival analysis (Figure 10D) further verified the clinical application potential of these treatment regimens, and throughout the experimental period, the body weights of the mice in all the treatment groups remained stable without significant fluctuations, indicating that the therapeutic agents used did not induce obvious systemic toxic reactions (Figure 10E). After the excision of the tumor in mice, tumor cells were implanted on the opposite side to construct the rechallenge model. TMO also has a very effective inhibitory effect on rechallenged tumors (Figure 10F). Given that multiple serum cytokines play key roles in regulating immune responses, we quantified the levels of major immunomodulatory cytokines in the serum via enzyme‐linked immunosorbent assay (ELISA). As shown in Figure 10G‒J, TMO treatment significantly upregulated the expression levels of antitumor cytokines, confirming that TMO treatment can effectively reverse the tumor immunosuppressive microenvironment and activate a systemic antitumor immune response. Effector memory T cells (T_EM_) play a critical role in suppressing tumor recurrence. In this study, we analyzed the infiltration levels of T_EM_ in blood. As shown in Figure 10K, TMO treatment significantly increased the proportion of T_EM_, which provides an important cytological explanation for its remarkable anti‐recurrence efficacy. TMO treatment significantly reduced the proportion of Eomes^+^ T_CM​_ cells (Figure 10L) while increasing T‐bet^+^ T_CM_​ cells (Figure 10M), indicating a shift toward an effector‐like memory phenotype. Over 28 days, T_EM_​ cells remained dominant in peripheral blood (Figure 10N), whereas both T_CM_​ and T_EM_​ cells persisted stably in lymph nodes (Figure 10O), supporting sustained memory responses under TMO treatment. As shown in Figure S19, we have supplemented the TMO treatment experiment for the HER2‐positive luminal‐like TUBO tumor therapy. TMO has a good anti‐tumor effect on TUBO tumors. Thus, it is speculated that our treatment method is applicable to various breast cancer subtypes and other types of tumors.

## 3 Conclusion

In summary, MnO_x_ nanozymes with precisely controllable valence states were successfully fabricated via electrochemical valence regulation. Our findings clearly demonstrated that the OXD activity of MnO_x_ nanozymes does not increase monotonically with the valence state of Mn. Instead, the medium‐valence sample 2O_V‐_MnO_2_, characterized by approximately 19.1% oxygen vacancies and a Mn d‐band center at −1.5 eV, exhibited optimal oxidase activity, thereby serving as the core material for subsequent antitumor applications. The TMO nanoformulation, constructed by encapsulating the optimized MnO_x_ nanozymes with TCM, achieved synergistic integration with multiple antitumor advantages. On the one hand, TCM endows TMOs with efficient tumor‐targeting capability, enabling them to induce ICD through OXD catalytic therapy. On the other hand, TMO can activate the cGAS‒STING pathway and T‐cell‐mediated tumor‐killing function. More innovatively, TMO integrates PD‐L1 antagonism, which effectively addresses the critical drawback that STING activation induces excessive PD‐L1 expression and thereby impairs T‐cell function. Comparative experiments confirmed that compared with PMO, TMO has superior tumor‐killing efficacy and stronger posttreatment recurrence inhibition.

Future research directions include elucidating the molecular mechanism by which MnOx modulates the d‐band center to optimize oxidase activity through advanced in situ characterization and theoretical calculations, extending the TCM coating strategy to various nanozymes and therapeutic agents for multimodal therapy, and combining TMOs with immunotherapies such as CAR‐T therapy to improve the treatment of metastatic tumors; nevertheless, the clinical translation of TMOs still faces major challenges, including scalable and controllable synthesis of uniform M‐MnOx, standardized preparation and preservation of functional PD‐1‐intact T lymphocyte membranes, guaranteed batch consistency of TMOs’ physicochemical and biological properties, and potential immune rejection caused by allogeneic cell membranes, which can be addressed by adopting autologous T‐cell membranes for personalized therapy, while long‐term biocompatibility and biosafety evaluation in large animal models is also essential for future clinical application.

## 4 Experimental Section

Experimental details are provided in the Supporting Information.

## Conflicts of Interest

The authors declare no conflicts of interest.

## Supporting information




**Supporting File**: exp270224‐sup‐0001‐SuppMat.docx.
