# Supplementary material for: Electrochemical Valence‐Regulated Biomimetic Nanozymes for Breast Cancer Metabolism Inhibition and Potentiated Catalytic Immunotherapy
Source: Exploration (Beijing). 2026 Sep 17;6(5):70224. doi: 10.1002/exp2.70224 (PMC13586063; doi:10.1002/exp2.70224)
Supplement: Supplementary file 1 [file EXP2-6-70224-s001.docx]

Supporting Information for

Electrochemical Valence-Regulated Biomimetic Nanozymes for Breast Cancer Metabolism Inhibition and Potentiated Catalytic Immunotherapy

**Experimental Section**

**Materials**

**The** ELISA kits DiO (DiOC18(3)) and Dil (DiIC18(3)) were purchased from Beijing Solarbio Science & Technology Co., Ltd. (China). All of the aqueous solutions were prepared using purified deionized (DI) water purified with a purification system (Direct-Q3, Millipore, USA). The other reagents used in this work were purchased from Sinopharm Chemical Reagent (China) and Aladdin-Reagent (China).

**Preparation of MnO_x_ (MO) samples with different oxidation states on FTO**

The MnO_x_ samples were prepared via a three-electrode electrochemical deposition system with an FTO substrate as the working electrode, a platinum foil as the counter electrode, and a Ag/AgCl electrode as the reference. Fluorine-doped tin oxide (FTO) conductive glass used in this study was purchased from Huanan Xiangcheng Technology Co., Ltd. It has a sheet resistance of approximately ~8 Ω sq^-1^, a thickness of about ~2.2 mm, and a transmittance higher than 80% in the visible light range. Prior to use, the FTO glass was ultrasonically cleaned in acetone, ethanol, and deionized water for 15 min each, and then dried in a vacuum oven. All the electrochemical measurements were performed on a Chenhua CHI760E electrochemical workstation. A deposition solution was prepared by dissolving 5 mmol of Mn(NO_3_)_2_ and 5 mmol of KNO_3_ in 50 mL of deionized water under thorough stirring to obtain a homogeneous and transparent electrolyte. The scan rate for cyclic voltammetry (CV) measurements was set to 100 mV s^-1^, with a potential window ranging from −2 to 2 V (vs. Ag/AgCl). During the potentiostatic deposition process, the electrolyte was maintained under quiescent conditions (without stirring) to avoid external disturbances affecting the deposition behavior. Potentiostatic deposition was conducted at 0.7 V, 1.1 V, 1.5 V, and 1.9 V to obtain MnO_x_ films with low-valence (L-MnO_x_), medium-valence (M-MnO_x_), high-valence (H-MnO_x_), and very-high-valence (VH-MnO_x_) states, respectively, with each deposition lasting 20 min. After deposition, the working electrodes were removed, rinsed gently with deionized water, and dried in a vacuum oven at 60 °C. Finally, the MnO_x_-coated FTO substrate was immersed in ethanol and subjected to brief ultrasonication (1–2 s) to detach the film from the substrate, and the collected material was dried to obtain dispersed MnO_x_ powders, which were subsequently used for structural characterization and performance evaluation. The extremely short ultrasonication time and mild mechanical effect ensure that the Mn valence states and oxygen vacancy content are well preserved during this delamination process.

**DFT calculations**

Density functional theory (DFT) calculations were performed via the Vienna Ab initio Simulation Package (VASP) with the projected augmented wave (PAW) method^[1]^. The exchange‒correlation functional was treated via the generalized gradient approximation of Perdew, Burke, and Ernzerhof (GGA-PBE)^[2]^. To account for the strong electron correlation in the Mn 3d orbitals, the DFT+U approach with an effective Hubbard parameter Ueff = 4.5 eV was applied^[3]^. A plane-wave energy cutoff of 420 eV and a 4 × 4 × 1 Monkhorst-Pack k-point mesh were used. The convergence criteria for total energy and atomic forces were set to ^10–5^ eV and 0.05 eV/Å, respectively.

For each MnO_2_ slab considered in this study, the energy difference associated with the O₂-bound configuration was calculated Eref = E(O_2_/MnO_2_) - E(MnO_2_), where MnO_2_ in this expression denotes the corresponding pristine or oxygen-vacancy-containing MnO_2_ slab. E(O_2_/MnO_2_) and E(MnO_2_) represent the total energies of the optimized O_2_-bound system and the corresponding slab without adsorbed O_2_, respectively. Note that the Eref is used only to compare the relative stabilization of O_2_-bound configurations across the models under the same computational setup.

**Electrochemical measurements**

Electrochemical measurements were performed via an electrochemical analyzer (CHI760E, CH Instruments, Inc.) with a three-electrode configuration. The prepared samples, a Ag/AgCl electrode, and a Pt foil served as the working, reference, and counter electrodes, respectively. Electrochemical impedance spectroscopy (EIS) was conducted at the open-circuit potential with an AC amplitude of 5 mV over a frequency range from 0.1 Hz to 100 kHz in a 1.0 M phosphate buffer solution (pH 6.5). All potentials were converted to the reversible hydrogen electrode (RHE) scale via the equation E_Ag/AgCl_ + 0.0591 × pH + 0.197.

**Materials characterization**

The crystalline structure was analyzed by X-ray diffraction (XRD, Rigaku Smartlab SE, Japan) using Cu Kα radiation (λ = 1.5406 Å) at 40 kV and 30 mA with a 2θ scanning range of 10–80°. X-ray photoelectron spectroscopy (XPS) measurements were performed on an ESCALAB 250Xi spectrometer (Thermo Fisher Scientific) with Al Kα radiation, and all binding energies were calibrated to the C 1 s peak at 284.8 eV. Transmission electron microscopy (TEM) and high-angle annular dark-field scanning transmission electron microscopy (HAADF-STEM) were performed via a JEM-F200 microscope (JEOL Ltd., Japan) equipped with a field emission gun operating at 200 kV. X-ray absorption fine structure (XAFS) measurements at the Mn K-edge were conducted at beamline BL14W1 of the Shanghai Synchrotron Radiation Facility (SSRF). The XAFS data were processed and analyzed via the Demeter software package. The spectra were normalized via Athena, and extended X-ray absorption fine structure (EXAFS) fitting was performed in R space via Artemis with k²-weighted Fourier transforms of the χ(k) function.

**Preparation and characterization of T-cell membrane (TCM)-coated MO (TMO), platelet membrane (PM)-coated MO (PMO) and erythrocyte membrane (RM)-coated MO (RMO)**

TCM, PM and RM were obtained according to previous methods^[4]^. Aqueous solutions of MO (0.2 mg/mL, 1 mL) and 1 mg TCM were then repeatedly coextruded through 200 nm pores by using a physical extruder (‌Avestin). The resulting TMO particles were centrifuged and washed with PBS several times to remove the excess TM. PMO and RMO were prepared in the same way as TMO.

Protein expression was determined by western blotting. The zeta potential was measured via DLS. The morphology of the synthesized materials was observed via field-emission TEM (JEM-F200). The thickness of the nanomaterials was determined via an atomic force microscope (Dimension Edge, Bruker).

**Cancer cell line culture**

The 4T1 and TUBO mouse breast cancer cell line was obtained from the Cell Bank of the Chinese Academy of Sciences and incubated in Dulbecco's Modified Eagle's Medium (DMEM) and RPMI-1640 medium (Gibco), respectively, supplemented with 10% fetal bovine serum, 100 U/mL penicillin G sodium and 100 μg/mL streptomycin sulfate in a humidified atmosphere containing 5% CO_2_ at 37 ℃.

**Animal tumor models**

Female BALB/c mice aged 5–6 weeks were purchased from Vital River Company (Beijing, China). BALB/c mice were subcutaneously injected with 2 × 10^6^ 4T1 cells in the right flank to construct a tumor model. All animal procedures were performed in accordance with the guidelines for the Care and Use of Laboratory Animals of the Ministry of Health in the People’s Republic of P. R. China and approved by the Animal Ethics Committee of Guangxi Medical University (2025-KYL(076)).

**Mn release study**

Ten milliliters of TMO containing 1 mg of MO was added to the culture dish. To investigate the effect of pH on the release behavior of Mn, a Mn release experiment was initially performed at different pH values (pH=6.5 or pH=7.4) for 48 h. At appropriate time points, 100 μL samples were collected for ICP‒MS measurements.

***In vitro* cancer cell internalization study**

4T1 cells were seeded in 24-well plates and cultured for 12 h. Then, 100 μL of Dil-labeled RMO, PMO, TMO or TMO+anti-PD-L1 antibody (containing 0.1 mg MnO_2_) was added to the medium. Then, the cells were incubated for 1 h or 4 h at 37 °C and 5% CO_2_ and washed with PBS three times. The cells were harvested, stained with DAPI, imaged via a fluorescence microscope (IX81, Olympus, Japan) and sorted via a flow cytometer (Beckman-Coulter, USA).

For Co-immunoprecipitation, TMO were mixed with free PD-L1 protein (MedChemExpress). The subsequent combined protein complex were subjected to immunoprecipitation with anti-PD-L1 antibody (Abcam) or normal IgG (Abcam) and protein A-Sepharose. Immune complexes were washed and subjected to immunoblotting with anti-PD-L1 (Abcam) or anti-PD-1 (Abcam) antibodies.

**Protein corona formation**

To detect the protein corona formation on TMO and bare nanoparticles (MO), equal MO amounts (containing 1 mg MO) of nanoparticles were separately suspended in 1 mL of fresh mice blood, followed by incubation at 37 °C with gentle shaking at 120 rpm for 30 minutes to allow sufficient protein adsorption. After incubation, the mixtures were centrifuged at 12,000 rpm and 4 °C for 20 minutes to precipitate the nanoparticles with adsorbed protein corona, the supernatant was carefully discarded to remove unbound free proteins, and the precipitates were washed three times with pre-cooled phosphate-buffered saline (PBS, pH 7.4) by centrifugation under the same conditions to eliminate loosely bound soft corona proteins. Subsequently, the protein concentration in the collected precipitates was determined using the BCA protein assay kit according to the manufacturer’s instructions, and the protein content per milligram of nanoparticles (μg protein/mg MO) was calculated to evaluate the protein corona adsorption capacity of TMO and MO.

**OXD catalytic activity and steady-state kinetic assays of M-MnO_x_**

The OXD catalytic activity of different MnOx nanoenzyme was evaluated using 3,3’,5,5’-tetramethylbenzidine (TMB) as the chromogenic substrate. Briefly, 3.0 mL of PBS (pH = 6.5) containing TMB (1 mM), and nanoenzyme (10 mg/mL) was incubated at room temperature (25 °C) for different time (1,3,5 minutes). After incubation, the UV-vis absorption spectra of the reaction solutions were recorded using a UV-vis spectrophotometer.

This property was also evaluated using ABTS as the chromogenic substrate. Briefly, ABTS solution was prepared in buffer solutions with different pH values of 5, 6, and 7. The higher activity nanoenzyme, e.g. M-MnOx, was chosen for evaluation. The oxidation of ABTS by M-MnOx produced green ABTS·^+^ that can show a characteristic spectrum with characteristic peak at 420 nm.

For steady-state kinetic assays, the catalytic activity of M-MnOx was further investigated. To determine the kinetic parameters, M-MnOx suspension was added to HAc-NaAc buffer solution (pH = 6.5) containing different concentrations of TMB. The concentration of M-MnOx was kept at 15 mg/mL in all kinetic assays. The absorbance of oxTMB was monitored at 652 nm using a Flex station3 microplate reader at different reaction times. The catalytic parameters were calculated by fitting data to the Michaelis-Menten equation, the experiments were repeated three times.

$$v=\frac{V_{max}\cdot[S]}{K_{M} + [S]}$$

where v is initial velocities. *V_max_* is the maximum rate of conversion, [S] is the substrate concentration and *Κ*_M_ is Michaelis constant.

***In vivo* pharmacokinetics and distribution study**

BALB/c mice (n = 3) received an intravenous (i.v.) injection of 100 μL of PBS containing MO, PMO or TMO (with an equivalent MO dose of 20 mg/kg). At different time points after the injection (i.e., 0.5, 1, 2, 4, 8, 24, and 48 h), 20 μL of blood was collected from the tail vein, dissolved in 1 M HCl and then centrifuged at 10000 rpm for 10 min. Finally, the supernatants were collected, and the Mn concentration was quantitatively analyzed via ICP‒MS as described above. At 7 14 and 28 days post-injection, mice were euthanized and major organs including liver, kidney, and brain were collected. The collected organs were digested, and the manganese (Mn) content was measured using inductively coupled plasma mass spectrometry (ICP-MS).

To study the biodistribution of TMO in various organs, BALB/c mice bearing 4T1 tumors (n = 3) received an intravenous (i.v.) injection of 100 μL of PBS containing MO, PMO or TMO (with an equivalent MO dose of 20 mg/kg). The main organs (heart, liver, spleen, lung and kidney) were removed at predetermined time points (6 h, 12 h and 24 h after the injection) and digested with aqua regia to study the biodistribution of the nanoparticles. After evaporation to dryness, the samples were redissolved in ultrapure water and measured via ICP‒MS.

***In vitro* ROS generation**

The intracellular generation of •O₂⁻ was detected via a DHE detection kit (Beyotime Biotech Inc). Afterwards, the cells were incubated for 5 different groups: (1) PBS; (2) TCM; (3) MO; (4) PMO; and (5) TMO. The MO concentration was 100 μg/mL in groups 3, 4 and 5. Then, the DHE detection kit was added. Then, the cells were detected under a fluorescence microscope (IX81, Olympus, Japan). The fluorescence intensity was measured with ImageJ software.

***In vitro* apoptosis detection**

Apoptosis was assessed by plating 4T1 cells in 6-well plates and treating them with 5 different groups: (1) PBS; (2) TCM; (3) MO; (4) PMO; and (5) TMO. The MO concentration was 100 μg/mL in groups 3, 4 and 5. After 12 h, the cells were harvested with EDTA-free trypsin, and annexin V-FITC/PI (Beyotime Biotechnology Co., Shanghai, China) was used to analyze apoptosis via flow cytometry.

**Detection of marker proteins involved in the cGAS‒STING pathway**

4T1 cells were seeded in 6-well plates and treating them with 6 different groups: (1) PBS; (2) TCM; (3) MO; (4) PMO; (5) TMO; and (6) TMO + STING inhibitor C178 (2 μM). The MO concentration was 100 μg/mL. After 12 hours of incubation, the cells from each group were collected, protein lysis solution was added to extract the total cell protein, and the protein was quantified according to the instructions of the BCA assay kit (Elabscience Biotechnology Co., Ltd., China). After electrophoresis, the separated proteins were transferred to PVDF membranes. After transfer, the PVDF membranes were blocked with 5% non-fat milk and diluted primary anti-cGAS antibody (Bioss, China), anti-phospho-IRF3 antibody (Bioss, China) or anti-phospho-STING antibody (Cell Signaling Technology, USA) solution (diluted at the manufacturer’s recommended dilution) was added overnight. After washing, the secondary antibody was added, and the samples were exposed, developed, and filmed.

**Immunogenic cell death (ICD) and PD-L1 detection.**

4T1 cells (1.5 × 10^5^ per well) were seeded in a 12-well plate for 12 h. Then, the cells were incubated with 6 different groups: (1) PBS; (2) TCM; (3) MO; (4) PMO; (5) TMO or (6) TMO+NAC (1 mM). The MO concentration was 100 μg/mL. For ICD detection, the cells were washed with PBS three times, fixed with 4% PFA for 10 min. After being washed with PBS three times, the cells were blocked with 10% FBS and incubated with an anti-calreticulin antibody, anti-HMGB1 antibody or anti-PD-L1 antibody (Bioss) and a fluorescently labeled secondary antibody for 30 min. The cells were washed with PBS three times and then stained with DAPI for 20 min. Finally, the cells were washed with PBS three times and observed via CLSM. The expression of PD-L1 was also detected by western blot.

To quantify the amount of HMGB1 and ATP released into the medium, the medium was collected after the cells were treated with the materials. Then, 20 μL of medium was used for ELISA detection or ATP detection kit (Elabscience Biotechnology Co., Ltd., China). The levels of secreted IFN-β in the samples were tested with ELISA kits (Guangzhou Ruiao Biotechnology Co., Ltd., China).

***In vitro* marrow-derived dendritic cell (BMDC) maturation study**

BMDCs were isolated from the bone marrow of 8-week-old BALB/c mice. For the BMDC maturation assay, 1 × 10^5^ 4T1 cells were treated by the above groups and then cocultured with 1 × 10^6^ BMDCs in the transwell culture system. Then BMDCs were stained with anti-CD80, and anti-CD86 (Biolegend). Then, the BMDCs were stained with fluorescence conjugated anti-CD11c, anti-CD80 and anti-CD86 antibodies (BD Biosciences, USA). Finally, the cells were analyzed by flow cytometer (Beckman‒Coulter, USA). The secretion levels of cytokines, including TNF-α, IL-6, and IFN-β, in the samples were tested with ELISA kits (Guangzhou Ruiao Biotechnology Co., Ltd., China).

**Evaluation of anti-tumor efficacy and biosafety of different doses of TMO in 4T1 tumor-bearing mice**

Briefly, 4T1 tumor-bearing BALB/c mice were randomly divided into five groups with five mice per group. When the tumor volume reached approximately 100 mm³ mice were treated with PBS or TMO at doses of 5 mg/kg, 10 mg/kg, 20 mg/kg or 40 mg/kg via intravenous injection every three days for a total of six administrations. Tumor volume was measured every two days using a vernier caliper and calculated by the formula volume = length × width² / 2. At the end of the experiment all mice were euthanized, and tumors were dissected and weighed. Blood samples were also collected to separate serum for the measurement of kidney function markers including CRE and BUN as well as liver function markers including ALT, AST, and ALP using commercial clinical assay kits.

***In vivo* antitumor therapy**

Female BALB/c mice aged 5–6 weeks were purchased from Vital River Company (Beijing, China). BALB/c mice were subcutaneously injected with 2 × 10^6^ 4T1 cells into the right flank on day -7. The mice were divided randomly into 5 different groups: (1) PBS; (2) TCM; (3) MO; (4) PMO; and (5) TMO. The MO dose was 20 mg/kg. The treatment was conducted on day 1 and day 3. The body weights and tumor volumes of the mice in all the groups were monitored every 3 days. A caliper was used to measure the tumor length and width, and the tumor volume was calculated according to the following formula. Tumor volume = tumor length × tumor width^2^/2. After 15 days of treatment, the mice were sacrificed. The tumor tissues were weighed, fixed in 4% neutral buffered formalin, processed routinely into paraffin, and sectioned at 4 μm. Then, the tumor sections were subjected to H&E staining, pimonidazole, mPTP, TUNEL, anti-p-STING and anti-PD-L1 antibodies and fluorescence-labeled secondary antibodies and finally examined via fluorescence microscopy (IX81, Olympus, Japan). The mouse lymph nodes (LNs) were immediately removed for immune analysis. The frequency of DC maturation in the LNs and T cell content in tumor tissues were then examined by flow cytometry after immunofluorescence staining with fluorescence-conjugated antibody. Mouse tumors were collected for ELISA (for TNF-α, IL-6, IFN-γ and IL-12p70) analysis (Elabscience Biotechnology Co., Ltd., China).

To assess the toxic side effects after TMO treatment, multiple sets of the above experiments were conducted. Within 28 days after TMO injection, HE histology of major organs and serum liver- and kidney-function markers were evaluated every 7 days. After injecting PBS and TMO for 28 days, BMDCs and CD8^+^ T lymphocytes from the peripheral blood of the mice were collected and stimulated *in vitro* with LPS (1 μg/mL for 24 hours) and CD3/CD28 beads respectively, to detect the cell functions.

We used TUBO cells to construct animal models in a similar manner as described above to evaluate the therapeutic effect of TMO on various types of breast cancer.

**Inhibition of tumor recurrence and rechallenge**

Female BALB/c mice aged 5–6 weeks were purchased from Vital River Company (Beijing, China). BALB/c mice were subcutaneously injected with 2 × 10^6^ 4T1 cells into the right flank at -13 days. The mice were divided randomly into 5 different groups: (1) PBS; (2) TCM; (3) MO; (4) PMO; and (5) TMO. The MO dose was 20 mg/kg. The treatment was conducted on the 3rd and 1st days. After the primary tumors were resected, the mice were rechallenged a secondary 4T1 tumor on the left hind leg. The growth of rechallenged secondary tumors and recurrent primary tumors were recorded at certain time intervals. The body weights and tumor volumes of the mice in all the groups were monitored every 3 days. A caliper was used to measure the tumor length and width, and the tumor volume was calculated according to the following formula. Tumor volume = tumor length × tumor width^2^/2. After 15 days of observation, the mice were sacrificed. The secretion of proinflammatory cytokines (TNF-α, IFN-γ, IL-6 and IL-12p70) in the serum after different treatments was measured via ELISA (Elabscience Biotechnology Co., Ltd., China). The proportion and function of CD8^+^ T cells and memory T cells in the blood were analyzed using flow cytometry.

**Statistical analysis**

All statistical data were processed in GraphPad Prism (version 8.0 for Windows) by Student’s t-test, log-rank test, one-way ANOVA, or two-way ANOVA. *Indicates significance at *p* < 0.05, ** indicates significance at *p* < 0.01, *** indicates significance at *p* < 0.001.

**Supplementary figures**

**Figure S1.** The UV-vis absorption spectra for MnOx nanozyme-catalyzed oxidation of ABTS under different pH.

**Figure S2**. XRD spectrum of M-MnO_x_.

| **Sample** | **ΔE_0_**/eV | **Scatter path** | **N** | **R/**Å | **σ^2^/**Å^2^ |
| --- | --- | --- | --- | --- | --- |
| M-MnO_x_ | -9.7 | Mn-O | 4.7 | 1.89 | 0.005 |
|  |  | Mn-Mn | 3.6 | 2.89 | 0.005 |

**Table S1.** EXAFS fitting parameters at the Mn K-edge for M-MnO_x_.

ΔE_0_: inner potential correction; N: coordination number; R: bond distance; σ^2^: Debye–Waller factor; S_0_^2^ was fixed to 1.0 for the samples. The fitting range is 2 < k < 11.5 Å^-1^, 1 < R < 3 Å.

**Figure S3.** Bader charge calculations of MnO_2_ and 2Ov-MnO_2_.

| **Sample** | **Mn** | **O** |
| --- | --- | --- |
| MnO_2_ | +1.84 | -0.92 |
| 2Ov-MnO_2_ | +1.82 | -0.93 |

**Table S2.** The average oxidation states of Mn and O obtained from Bader charge analysis.

**Figure S4.** TEM images of the (A) PM (scale bar: 200 nm) and (B) PMO (scale bar: 200 nm). (C) Diameter and (D) zeta potential of MO, PM and PMO. The data are displayed as the mean ± SD (n = 3).

**Figure S5.** Protein corner content adsorbed on MO and TMO over time. Data represent mean ± SD (n = 3).

**Figure S6.** The Co-immunoprecipitation assay with anti-PD-L1 antibody (or IgG) showed the binding of TMO to PD-L1, followed by immunoblotting of PD-1 and PD L1.

**Figure S7.** Fluorescence images of 4T1 cells incubated with Dil labeled TMO without or with anti-PD-L1 antibody for 1 h (Blue: DAPI; red: Dil).

**Figure S8.** Analysis of ATP released from 4T1 cells after different treatments. The data are displayed as the mean ± SD (n = 3). Statistical significance was calculated via one-way ANOVA with Tukey’s test: ***p < 0.001.

**Figure S9.** CLSM images of CRT (red) expressions in 4T1 cells after different treatments. NAC: 1mM.

**Figure S10.** Western blot assay of p-STING activation in different treated 4T1 cells. A potent and selective inhibitor of STING (C178, 2 μM) was added to the TMO group.

**Figure S11.** Detection of PD-L1 expression of cancer cells after indicated treatment via western blot analysis.

**Figure S12.** Changes in 4T1 tumor volume and tumor weight after TMO treatments with different dosages. The data are shown as the mean ± SD (n = 5). Statistical significance was calculated *via* one-way ANOVA with Tukey’s test: ^***^p < 0.001, ns: no significance.

**Figure S13.** The kidney function markers BUN and CRE and the liver function markers ALT, ALP, and AST were measured after i.v. injection of TMO with different dosages. The data are displayed as the mean ± SD (n = 5). Statistical significance was calculated via one-way ANOVA with Tukey’s test: ^*^p < 0.05, ^**^p < 0.01, ns: no significance.

**Figure S14.** HE staining of major organs on day 7, 14, and 28 post i.v. injection of TMO.

**Figure S15.** The kidney function markers BUN and CRE and the liver function markers ALT, ALP, and AST were measured in TMO group on day 7, 14, and 28 post i.v. injection of TMO. The data are displayed as the mean ± SD (n = 5). Statistical significance was calculated via one-way ANOVA with Tukey’s test. ns: No Significance.

**Figure S16.** After injecting PBS and TMO for 28 days, BMDCs and CD8^+^ T lymphocytes from the mice were collected and stimulated *in vitro* with LPS (1 μg/mL 24h) and CD3/CD28 beads respectively, for the purpose of detecting the cell functions. The data are displayed as the mean ± SD (n = 3).

**Figure S17.** The Mn content of the main organs at different time points after TMO injection. The data are displayed as the mean ± SD (n = 3).

**Figure S18.** Pimonidazole (PIMO) and mPTP staining of tumor sections after the indicated treatments.

**Figure S19.** Changes in TUBO tumor volume and tumor weight after indicated treatments. The data are shown as the means ± SD (n = 5).

**References**

[1]	a)G. Kresse, J. Hafner, *Phys Rev B Condens Matter* **1994**, 49, 14251; b)P. E. Blöchl, *Phys Rev B Condens Matter* **1994**, 50, 17953.

[2]	J. P. Perdew, K. Burke, M. Ernzerhof, *Phys Rev Lett* **1996**, 77, 3865.

[3]	B. Tian, S. Ma, J. Guo, Y. Zhao, T. Gao, X. Jiang, *Applied Surface Science* **2022**, 601, 154162.

[4]	a)J. Su, H. Sun, Q. Meng, P. Zhang, Q. Yin, Y. Li, *Theranostics* **2017**, 7, 523; b)M. Zhang, L. Gu, R. Luo, D. Liu, J. Li, Z. Deng, X. Luo, X. Chen, S. Ning, *Materials Today* **2025**, 88, 272; c)X. Luo, Y. Zhou, K. Rao, J. Xiang, S. Ning, D. Zhu, G. Li, H. Chen, *Small* **2025**, e2411576.
